# Developing and Validating a Global Governance Framework for Health: A Delphi Consensus Study

**DOI:** 10.3390/ijerph23010138

**Published:** 2026-01-22

**Authors:** Kadria Ali Abdel-Motaal, Sungsoo Chun

**Affiliations:** Institute of Global Health and Human Ecology, American University, New Cairo 11835, Egypt; kkmotaal@aucegypt.edu

**Keywords:** global health governance, pandemic preparedness, WHO pandemic agreement, Delphi consensus study, cross-sectoral integration

## Abstract

**Highlights:**

**Public health relevance—How does this work relate to a public health issue?**

- The study addresses persistent global governance failures exposed during COVID-19, including inequity, weak coordination, and limited accountability mechanisms that undermine global health security.
- It evaluates the WHO Pandemic Agreement through an evidence-based governance lens, identifying operational gaps that directly affect preparedness and public health protection.

**Public health significance—Why is this work of significance to public health?**

- The GGFH Framework shifts pandemic preparedness from a health-sector focus to an integrated governance-for-health model, enabling coordinated action across health and non-health sectors.
- The GGFH Framework attempt to complement the implementation of the WHO Pandemic Agreement by addressing partially resolved areas, providing practical governance solutions to gaps in financing, accountability, equity, and cross-sectoral coordination.

**Public health implications—What are the key implications or messages for practitioners, policy makers and/or researchers in public health?**

- The GGFH offers policymakers a validated governance alternative that replaces fragmented, market-driven responses with a model based on global public goods, enforceable obligations, and shared accountability.
- A phased implementation approach is recommended, prioritizing foundational legal, leadership, and financing reforms to enable effective rollout of subsequent equity, accountability, and cross-sectoral measures.

**Abstract:**

Background: The COVID-19 pandemic exposed major deficiencies in global health governance, including fragmented authority, inequitable resource distribution, and weak compliance mechanisms. Although the WHO Pandemic Agreement (2025) addresses several of these gaps, significant operational and institutional challenges remain. This study aims to develop and empirically validate a Global Governance for Health (GGFH) Framework that strengthens leadership, financing, equity, and legal accountability across global, regional, and national levels. Methods: A three-round Delphi study was conducted. Thirty-one experts from diverse sectors, including public health, international law, economics, environment, and diplomacy, evaluated 32 structured governance statements across seven domains. Experts rated all statements using a 7-point Likert scale. Consensus was determined using a strict threshold median ≥ 6; SD ≤ 1.35; ≥75% agreement. Open-text comments were systematically reviewed through thematic analysis. All statements were systematically mapped to the WHO Pandemic Agreement articles to identify areas lacking operational clarity or enforceability. Results: All seven governance domains achieved consensus by Round 3. High agreement emerged on strengthening WHO leadership, implementing sustainable and equitable financing mechanisms, embedding LMIC representation, establishing legal preparedness and capacity-building, and integrating independent accountability tools. Correlation and interdependence analyses demonstrated that governance goals form an integrated, mutually reinforcing system, with financing, equity, and legal frameworks identified as core enablers of effective treaty implementation. Conclusions: The Delphi process validated a comprehensive and operational Global Governance for Health Framework. The GGFH complements the WHO Pandemic Agreement by addressing its unresolved governance, financing, and equity limitations and offers a structured roadmap to guide global pandemic preparedness and treaty implementation.

## 1. Introduction

The adoption of the WHO Pandemic Agreement (WHA 78.1) in May 2025 marked a pivotal moment in the evolution of global health governance. Developed in response to the structural failures of the global health governance system exposed by the COVID-19 pandemic, the agreement aims to institutionalize global solidarity, equity, and preparedness through a legally binding framework [1]. Despite this ambition, the pandemic exposed enduring limitations in existing systems, including fragmented authority, inequitable resource allocation, and a lack of effective compliance mechanisms, shortcomings that the WHO Pandemic Agreement addresses only in part [2,3,4].

Over the past two decades, the architecture of global health governance (GHG) has become increasingly pluralistic. Alongside states and multilateral institutions, a growing array of actors, including philanthropic foundations, public–private partnerships, and civil society, have assumed prominent roles in global health decision-making [5]. This shift has fostered what Kickbusch has termed “networked governance”: a model defined by interdependence and innovation yet characterized by blurred lines of accountability and authority [5]. During COVID-19, this diffusion manifested in overlapping mandates and operational tensions among entities such as the World Health Organization (WHO), Gavi (the Vaccine Alliance), the Coalition for Epidemic Preparedness Innovations (CEPI), and COVAX (the COVID-19 Vaccines Global Access Facility) [6,7].

The WHO Pandemic Agreement represents the most comprehensive institutional response to these governance failures to date. It introduces important provisions related to data sharing, sustainable financing, equitable access to countermeasures, and One Health integration [8]. However, comparative treaty analysis and policy reviews reveal that it still falls short of addressing deeper governance challenges. Provisions related to financing, enforcement, and cross-sectoral coordination remain general, and some mechanisms, such as the Pathogen Access and Benefit-Sharing (PABS) system, remain deferred to future annexes pending consensus. These omissions reflect a broader conceptual limitation in existing frameworks: a tendency to emphasize the “what” of global health, surveillance, financing, innovation, while under developing the “how” of authority, coordination, and accountability [9,10]. As a result, governance responses remain reactive, institutionally fragmented, and normatively aspirational [11].

In response to this persistent gap, this study proposes and empirically validates a novel Global Governance for Health (GGFH) framework. Grounded in existing theoretical approaches, including multilevel governance, network governance, complex adaptive systems, and principal-agent models, the framework is designed to operationalize key governance principles across structural, normative, and performance domains. Specifically, it integrates three interdependent dimensions:

1. Structural/institutional: configuration of actors, authority distribution, and coordination mechanisms.
2. Normative/ideational: principles such as equity, transparency, solidarity, and human rights.
3. Performance/outcome: effectiveness, legitimacy, and accountability in achieving global health goals.

Importantly, the COVID-19 crisis reaffirmed that health governance cannot be siloed. Pandemic risk is shaped not only by public health systems but by broader structures, trade regimes, intellectual property rules, global finance, armed conflict, and environmental degradation [4,12]. Addressing such risks requires governance frameworks that are integrated across sectors and adaptable to geopolitical realities.

To empirically validate the proposed GGFH framework, the study applies the Delphi method, a structured, iterative technique for gathering expert consensus on complex and uncertain issues [13,14,15,16]. A diverse panel of experts in global health, law, finance, policy, and cross-sectoral integration was engaged across three rounds. This process not only tested the framework’s conceptual robustness but also enabled refinement of its operational linkages and institutional priorities through structured feedback loops.

This Delphi study pursues three interlinked objectives:

1. To validate the relevance, coherence, and completeness of the proposed GGFH framework.
2. To refine its structural, normative, and performance indicators based on expert consensus; and
3. To develop a consensus-informed model that can guide implementation, evaluation, and reform of global health governance systems.

By synthesizing lessons from COVID-19, a systematic treaty analysis, and the collective judgement of a multidisciplinary expert panel, this study offers both conceptual advancement and practical guidance. The GGFH framework moves beyond critique to propose a validated, implementable model for global governance, one designed to enhance coordination, promote equity, and embed legal accountability at the heart of future pandemic preparedness.

## 2. Literature Review

The COVID-19 pandemic prompted renewed scrutiny of global health governance, exposing persistent structural gaps in coordination, equity, financing, and accountability within both national systems and international institutions. Numerous analyses have demonstrated that pandemic response effectiveness was shaped not only by health-system capacity, but also by political decision-making, global supply-chain interdependencies, governance fragmentation, and the absence of enforceable global norms [17,18]. These shortcomings have renewed calls for governance reforms capable of supporting the implementation of the WHO Pandemic Agreement (2025), particularly as the treaty remains largely normative and lacks operational clarity in several domains [19].

A critical conceptual distinction informs this study. Global health governance (GHG) traditionally refers to governance within the health sector, such as the WHO, ministries of health, and global health initiatives. In contrast, global governance for health (GGfH) recognizes that health outcomes are shaped by decisions made outside the health sector, including in trade, finance, intellectual property, environment, labour, and security, and as Kickbusch and colleagues contend, by broader global forces that necessitate governance arrangements extending across policy domains [20]. This broader framing is essential because pandemic risks and responses are shaped by multisectoral determinants that cannot be addressed through health institutions alone. COVID-19 demonstrated that health challenges cannot be governed in silos; instead, they require cooperation across economic, legal, political, diplomatic, environmental, and technological domains. This conceptual shift underpins the need for a holistic governance framework capable of coordinating actors beyond the traditional health sphere.

### 2.1. Governance Failures During COVID-19

COVID-19 revealed persistent failures in global governance structures, including inequalities in access to vaccines and diagnostics, poor coordination between multilateral institutions, and insufficient global financing for preparedness. A mixed-methods analysis by Abdel-Motaal and Chun synthesized empirical indicators and qualitative evidence to identify the systemic nature of these failures [17]. Their findings showed that inequitable distribution of medical countermeasures, fragmented leadership roles, and limited accountability mechanisms were central contributors to the uneven global response. These insights informed the development of Delphi domains related to leadership, financing, transparency, and operational feasibility.

### 2.2. Structural Weaknesses in Global Health Governance

A complementary scoping review by Abdel-Motaal et al. mapped the structural determinants that have historically undermined effective global pandemic governance [18]. The review identified chronic deficiencies in legal enforceability, multisectoral coordination, equitable resource allocation, and oversight mechanisms. Importantly, many of these weaknesses persisted despite successive reform attempts and are only partially addressed in the WHO Pandemic Agreement. These findings shaped Delphi domains concerning equity, financing, accountability, and cross-sectoral integration.

### 2.3. Theoretical Foundations Informing the Delphi Framework Development

To situate pandemic governance reform within established theoretical frameworks, three bodies of governance theory were incorporated.

#### 2.3.1. Multilevel Governance

Multilevel governance describes how authority is redistributed upward to global institutions, downward to regional bodies, and horizontally to networks, private actors, and civil society [21,22]. This concept clarifies the complex institutional landscape of pandemic preparedness, where actors such as the WHO, regional entities, financial institutions, and non-state actors interact across multiple levels. It provides the conceptual basis for Delphi statements relating to leadership distribution, regional coordination, and stakeholder engagement.

#### 2.3.2. Meta-Governance

Meta-governance refers to the governance of governance, how overarching rules, norms, and coordination mechanisms shape interactions among diverse actors [23]. Pandemic preparedness requires strong meta-governance functions, including standard-setting, monitoring, incentivisation, and accountability. These functions are central to proposals for strengthening WHO’s coordinating role and clarifying operational responsibilities under the Pandemic Agreement.

#### 2.3.3. Good Governance

Good governance frameworks emphasize transparency, participation, rule of law, accountability, and effectiveness as prerequisites for equitable outcomes [24]. During COVID-19, deficits in transparency, data sharing, and resource allocation directly contributed to inequitable outcomes, as shown in multiple analyses [17,18,25]. These principles guided the development of Delphi items concerning accountability mechanisms, equitable financing, and legal compliance.

Together, these theories provided the conceptual scaffolding for translating complex governance constructs into structured Delphi statements suitable for expert evaluation

### 2.4. The Paradox of Global Governance: Why It Is Needed, and Why Some Nations May Resist

The need for global governance for health arises from the transboundary nature of pandemic threats and the dependence of health security on coordinated surveillance, data sharing, supply-chain stability, and cross-sectoral policies. Yet, the same mechanisms that make collective action essential also make it politically sensitive. States may hesitate to participate due to concerns about sovereignty, unequal power dynamics, and distrust of multilateral institutions. This paradox, that pandemics require strong global cooperation precisely when political incentives may favour national autonomy, highlights the necessity of governance arrangements perceived as legitimate, fair, and mutually beneficial. Recent pandemic experiences have underscored that global threats can carry cascading societal and economic consequences, reinforcing the urgency of more coherent international cooperation.

### 2.5. Evidence from Past Reform Attempts

Previous assessments by the Global Preparedness Monitoring Board (GPMB) and the WHO Health Emergency Preparedness, Response and Resilience (HEPR) programme consistently identified gaps in global coordination, financing, and emergency leadership, many of which persisted during COVID-19 [26,27]. These evaluations revealed that reforms often remained aspirational and failed to translate into operational mechanisms. Their findings informed Delphi statements targeting enforceability, oversight, and financing architecture.

### 2.6. Treaty Analysis and Implementation Gaps

A document analysis of the 2025 WHO Pandemic Agreement highlighted articles that are normatively strong but operationally vague, particularly in areas of equity, financing, capacity-building, and accountability [18,19]. Mapping each Delphi statement to relevant treaty articles allowed systematic assessment of whether proposed governance reforms meaningfully support treaty implementation.

### 2.7. Contribution of This Study

Given the limitations of existing governance models, the unresolved challenges exposed by COVID-19, and the operational gaps in the Pandemic Agreement, there is a clear need for a more coherent, integrated governance framework. The contribution of this study is twofold:

1. It proposes a new Global Governance for Health (GGfH) framework that unifies structural, normative, and performance dimensions of governance in a manner not addressed by prior literature.
2. It empirically validates this framework through a Delphi process that synthesizes multidisciplinary expert consensus to define practical, evaluable governance functions.

This study therefore moves beyond descriptive analyses of governance failures and provides an actionable, consensus-based model designed to strengthen global coordination, equity, and accountability in future pandemics.

## 3. Delphi Methodology and Instrument Design

### 3.1. Overview

This Delphi study was undertaken to obtain expert evaluation of a proposed Global Governance Framework for Health (GGFH), developed to address long-standing and emergent gaps in global health governance. The framework builds upon the evolving concept of global governance for health, which acknowledges that health outcomes are shaped not solely within health systems, but through complex and dynamic interactions across multiple global domains. These include international trade, legal regimes, financial policy, diplomacy, environmental sustainability, and social equity.

Recognizing this interdependence, the composition of the Delphi panel was purposefully designed to reflect a wide spectrum of expertise. Participants were drawn not only from public health, epidemiology, and health systems, but also from relevant sectors such as international law, economics, finance, environmental governance, trade, and global diplomacy. This approach was designed to deepen analysis, promote cross-sectoral integration, and ensure the policy relevance of the findings.

### 3.2. Study Design

The Delphi method was selected for its suitability in fostering structured consensus among experts, particularly in domains marked by high uncertainty and cross-disciplinary complexity such as global health governance reform. Its core strengths, anonymity, iteration, controlled feedback, and statistical aggregation, helped minimize bias, avoid dominance by individual voices, and enable convergence over multiple rounds. These methodological principles are well-established in the Delphi literature [13,14,15,16].

A three-round, cross-sectional Delphi study design was used. Participants assessed the feasibility, relevance, and alignment of GGFH components with the WHO Pandemic Agreement (2025). Iterative rounds allowed refinement of statements and clarification of areas of convergence and divergence. The international and cross-sectoral composition of the panel added rigour by enabling a pluralistic assessment of the proposed framework.

The Delphi instrument was organized into seven domains, reflecting key pillars of global health governance:

1. WHO Leadership Role in Pandemic Governance.
2. Redefined Role of the WHO Regional Offices.
3. Sustainable and Equitable Financing.
4. Equity Mechanisms.
5. Accountability, Monitoring.
6. Cross-Sectoral Integration across Health and Non-Health Domains.
7. Legal and Policy Framework.

Participants were also asked to Assess the GGFH framework’s comprehensiveness and inclusivity.

Each domain served as the basis for a cluster of Delphi statements; a total of 32 structured statements was developed and distributed across the eight thematic domains (Supplementary Materials File S1). Each statement was assessed by participants using a 7-point Likert scale, measuring relevance and operational feasibility.

To ensure relevance and alignment with policy priorities, each Delphi statement was systematically mapped to corresponding articles of the WHO Pandemic Agreement, identifying areas where normative commitments lacked operational clarity or enforceability. This structured gap analysis is detailed in Supplementary File S2 “Comparing WHO Pandemic Agreement Articles Gaps vs. Delphi Statements”.

This study explicitly positions the WHO as the central actor in global health governance because it remains the only intergovernmental organization with a constitutional mandate to coordinate international health work, set normative standards, and lead global responses to public health emergencies under the International Health Regulations, 2005 [28,29]. While global health governance is increasingly pluralistic, with significant roles played by regional bodies, philanthropic organizations, and public–private partnerships, the WHO continues to serve as the legally recognized convener of multilateral cooperation in pandemics. The WHO Pandemic Agreement (2025) reinforces this role by assigning the Organization responsibility for stewardship, coordination, and monitoring of global preparedness and response [19]. Therefore, anchoring the Delphi assessment in the WHO framework is justified both legally and operationally, while still acknowledging the importance of complementary actors in a multi-level governance environment. The assumption of WHO leadership is thus not normative but grounded in existing international law, treaty structures, and state practice

### 3.3. Delphi Instrument Validation

The Delphi instrument was informed by a series of complementary studies that together ensured conceptual rigour, empirical relevance, and policy alignment. First, prior mixed-methods research [17] provided the empirical basis for identifying the governance challenges the Delphi sought to evaluate. Second, a recent scoping review [18] mapped the structural determinants and institutional gaps shaping global health governance, directly informing the thematic domains and clustering of Delphi statements. Third, theoretical models of global governance, including multilevel governance, meta-governance, and good governance frameworks, were incorporated to capture the structural, institutional, and political dimensions relevant to pandemic preparedness [21,22,23,24,25].

Fourth, evaluations of previous reform efforts, such as those conducted by the GPMB and the HEPR programme [26,27], were instrumental in shaping the Delphi statements. These assessments highlighted expert orientations regarding where reform efforts should be directed and ensured that the Delphi items reflected both the empirical lessons of past governance failures and the areas toward which informed expert consensus is most likely to converge.

Finally, a detailed document analysis of the WHO Pandemic Agreement (2025) [18,19] identified treaty provisions that remain aspirational, operationally vague, or insufficiently responsive to governance failures revealed during COVID-19. Each Delphi statement was systematically mapped to the relevant articles of the WHO Pandemic Agreement to identify where normative commitments remain operationally unclear (Supplementary Materials File S2).

Together, these five foundational sources ensured that the Delphi instrument was conceptually grounded, empirically informed, and directly relevant to strengthening the implementation of the WHO Pandemic Agreement.

Thematic clusters were created to group related statements, supported by explanatory notes to guide expert interpretation. To validate the instrument, a pilot was conducted with subject-matter experts. Feedback-informed adjustments in phrasing, reduced redundancy, and improved clarity. Each item included a 7-point Likert scale for structured rating and optional open-text fields for qualitative feedback.

### 3.4. Sampling Techniques

A purposive expert sampling approach was used, following established Delphi standards. A structured three-stage identification process ensured methodological rigour and minimized selection bias:

1. Long-list generation: A list of 186 potential experts was compiled using predefined criteria, including peer-reviewed contributions to global health governance, pandemic preparedness, international law, or global health financing; senior institutional roles (e.g., WHO, UN agencies, multilateral organizations, civil-society networks, academic institutions); and ≥10 years of relevant professional experience.
2. Screening: Candidates were assessed for thematic relevance, geographic diversity, and sectoral balance to avoid overrepresentation from any region, institution, or ideological orientation.
3. Shortlisting and invitations: Experts were invited based on independence from the research team and absence of ties to WHO bodies involved in drafting the Pandemic Agreement. Conflicts of interest were screened and documented. The anonymity of Delphi rounds further reduced dominance effects. This structured process aligns with best practice and the methodological intent of Delphi research, where informed judgement, not statistical representativeness, is the core objective [15].
4. Panel Size and Inclusion Criteria: The final panel comprised 31 experts, selected according to the following criteria:

- Professional expertise: ≥10 years in pandemic preparedness, global health law, international finance, diplomacy, or related fields.
- Academic contribution: Peer-reviewed publications or demonstrated policy impact.
- Applied experience: Roles in governance, negotiation, or institutional reform.
- Sectoral diversity: Representation from academia, government, multilateral agencies, civil society, and global health initiatives.
- Demographic considerations: Attention to gender and age diversity.
- Commitment: Participation across all Delphi rounds.
- Geographic Representation.

Recruitment combined direct invitations with snowball sampling to broaden regional and sectoral coverage while maintaining methodological control [30]. To enhance transparency and contextual interpretability, the Delphi panel included experts based in Uganda, Pakistan, Indonesia, Saudi Arabia, India, Palestine, Egypt, Greece, Croatia, the United Kingdom, France, Switzerland, the United States, South Africa, Lesotho, the Netherlands, Nigeria, and Sudan. This distribution ensured substantial participation from LMICs and from regions with diverse governance systems, health capacities, and pandemic experiences, an essential feature for evaluating global governance and equity considerations.

### 3.5. Delphi Rounds and Feedback Mechanism

A total of 65 experts were initially invited to participate in the Delphi process; 42 accepted the invitation, and 31 completed the Round 1 survey, resulting in a final expert panel of 31 participants. This corresponds to a response rate of 73%, exceeding standard methodological thresholds [15]. In Round 2, 30 of the original participants continued, reflecting a 96% retention rate, consistent with recommended Delphi benchmarks (≥70%) [18].

#### The Delphi Process Consisted of Three Iterative Rounds

Round 1: Experts rated 32 structured statements across eight governance domains, evaluating relevance, feasibility, and alignment with the WHO Pandemic Agreement (2025). This round included two additional modules: (1) a goal prioritization ranking the seven governance goals by strategic importance, and (2) an interdependence analysis assessing cross-domain influence.

Round 2: Participants received summary statistics (median, SD), anonymized peer feedback, and their prior responses, enabling reflection and optional revision of ratings.

Round 3: A focused rating round with 15 experts (50% of the original panel), exceeding the 30% threshold for valid subgroup Delphi rounds [16]. Revised statements, shaped by rounds 1 and 2 feedback, addressed issues of clarity, institutional roles, legal duplication, feasibility, and equity. Revised statements were presented alongside original versions and feedback summaries to promote transparency. Supplementary Materials File S3 provides a comparative table of original and revised items.

Ethical approval was obtained from the relevant review board, and electronic informed consent was secured from all participants.

### 3.6. Consensus Criteria

To evaluate expert agreement, this study incorporated both strict and flexible consensus thresholds.

- Strict consensus: Median ≥ 6, standard deviation (SD) ≤ 1.35, and ≥75% agreement.
- Flexible consensus: Median ≥ 5, SD ≤ 1.5, and ≥75% agreement.

These thresholds (see Table 1) align with 82–87% coverage under a normal distribution, balancing analytical rigour with broad expert inclusion 

### 3.7. Data Analysis Methods

#### 3.7.1. Quantitative Analysis

Quantitative data were analyzed using descriptive and inferential statistics: Measures of central tendency and dispersion (mean, median, standard deviation, and interquartile range) were calculated for each of the 32 statements across all rounds. Consensus achievement was evaluated using the predefined strict and flexible thresholds (see Section 3.6). Goals prioritization was analyzed using mean rank scores. Perceived interdependencies between governance domains were assessed using average influence ratings to determine how progress in one domain was seen to affect others. To assess coherence and inter-domain relationships, two correlation analyses were conducted:

(a) Score-Based Correlation

To assess the internal coherence of expert judgments across governance domains, a Pearson correlation matrix was computed using domain-level composite scores. These scores were generated by averaging individual Likert-scale responses across all validated statements within each thematic goal. The Pearson correlation coefficient (r) quantifies the strength and direction of linear relationships between domains, with values ranging from –1.0 (perfect negative correlation) to +1.0 (perfect positive correlation). This analysis helped determine whether experts viewed the governance goals as integrated components of a broader system, based on consistent patterns in scoring [13].

(b) Prioritization-Based Correlation

Spearman’s rank-order correlation coefficients were calculated to analyze expert prioritization patterns across the seven governance domains. Spearman’s method is particularly appropriate for ordinal data and was used to assess alignment in strategic vision among panel members [13,14].

#### 3.7.2. Qualitative Analysis

Open-ended responses accompanying each Delphi statement were thematically analyzed to complement the quantitative data. The process involved: Coding responses to identify recurring themes, conceptual insights, and operational concerns; Synthesizing suggestions to refine terminology, clarify institutional roles, and enhance feasibility; Highlighting dissenting or minority viewpoints that challenged assumptions or proposed alternative strategies. This analysis provided insight not only into consensus areas but also into the reasoning and divergence behind expert views.

#### 3.7.3. Triangulation of Quantitative and Qualitative Data

A mixed-methods triangulation strategy integrated statistical results with expert narratives to enhance interpretive depth. This approach ensured that both areas of agreement and contestation informed the evaluation and iterative refinement of the Global Governance for Health Framework (GGFH).

## 4. Results

### 4.1. Demographic Analysis

The final Delphi panel comprised 31 experts with diverse geographic, institutional, and disciplinary profiles. This diversity enhanced the panel’s epistemic legitimacy by integrating wide-ranging expertise and regional perspectives, a key strength of Delphi methodology in global health governance.

#### 4.1.1. Gender Distribution

The panel achieved near gender parity: 51.6% female (*n* = 16) and 48.4% male (*n* = 15), supporting deliberative legitimacy and aligning with SDG 5 on gender equality (Table 2).

#### 4.1.2. Age Distribution

Panellists were primarily senior professionals: 38.7% aged 50–60; 32.3% aged 45–50; 19.4% aged 60+; and 9.6% aged 30–35. This composition balanced institutional memory with emergent perspectives, combining historical insight with forward-looking thinking, (Table 2).

#### 4.1.3. Geographic and Institutional Representation

The panel achieved a global balance, with 60% of participants from low- and middle-income countries (LMICs) and 40% from high-income countries (HICs). This distribution was deliberate, grounded in principles of equity and representational justice, central concerns in global health diplomacy. Including LMIC voices ensured that perspectives from structurally disadvantaged regions were foregrounded in governance reform.

Institutional representation was similarly diverse: 37% from academia, 30% from international organizations, 13% from government, and 20% from NGOs, the private sector, or research institutes. This cross-sectoral composition enhanced the Delphi process by surfacing blind spots and enriching deliberation through multiple institutional lenses.

#### 4.1.4. Disciplinary Expertise

To reflect the cross-cutting nature of pandemic governance, the expert panel was deliberately composed of individuals from diverse professional disciplines. Participants were categorized into four primary areas based on their institutional affiliations and core expertise:

- Public Health and Health Systems (53%): Including epidemiologists, implementation researchers, and policymakers focused on public health.
- Economics and Finance (23%): Experts in economic policy and financial governance, particularly from global institutions.
- Governance, Law, and International Policy (17%): Specialists in international law, diplomacy, and treaty development.
- Environment, One Health, and Cross-Sectoral Collaboration (7%): Experts at the intersection of environmental, animal, and human health systems.

**Figure 1:**
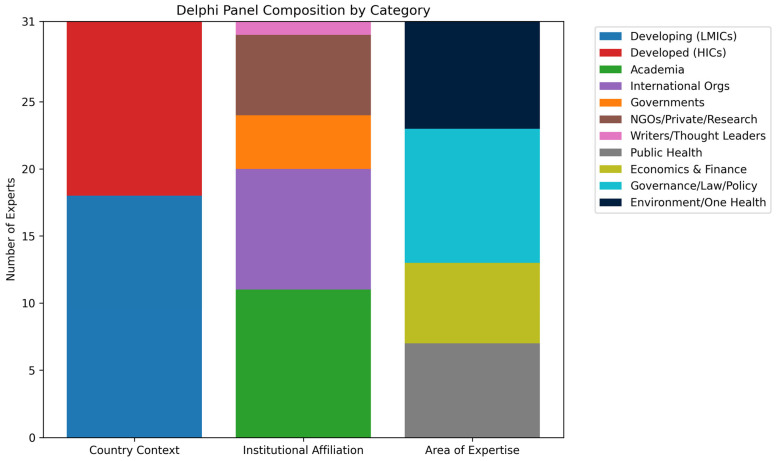
Delphi Panel Decomposition.

This disciplinary diversity ensured a holistic assessment of the framework from technical, legal, financial, and ethical perspectives. Visual summaries of participants’ country contexts, institutional affiliations, and expertise areas are provided in Figure 1 highlighting the intentional integration of varied knowledge systems and actor typologies, key principles in inclusive governance reform.

### 4.2. Strategic Prioritization and Inter-Goal Dynamics

#### 4.2.1. Governance Goals Prioritization

Method and Rationale for Prioritization: To complement the content evaluation of governance domains, in Delphi Round 1, participants were asked to rank the seven GGFH framework goals by strategic importance. This exercise extended beyond Likert-scale ratings to capture perceptions of urgency, implementation sequencing, and catalytic potential factors that Delphi methods use to assess not only feasibility and relevance but also institutional and political salience in global health governance [14].

**Figure 2:**
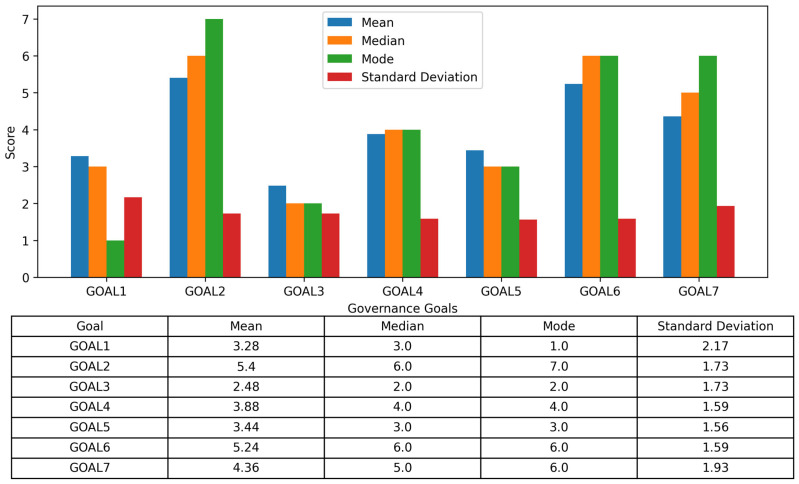
Descriptive Statistics for Goal Prioritization (Source: Delphi Round 1).

Descriptive Results of Goal Rankings: Figure 2 presents expert rankings across seven governance goals, including mean, median, mode, and standard deviation. Lower means indicate higher priority.

Strategic Preferences and Interpretive Patterns: Experts prioritized goals that function as systemic enablers of the wider governance architecture. Goal 3, “Sustainable and Equitable Financing,” received the highest priority (mean = 2.48), reflecting consensus that predictable, equity-sensitive financing underpins all other reforms. This aligns with calls to operationalise Article 19 of the WHO Pandemic Agreement early to support the treaty’s implementation. Goal 1, “WHO Leadership,” also ranked highly (mean = 3.28), consistent with institutionalist arguments that strong central coordination is essential in fragmented governance environments [31].

Mid-priority goals included Goal 4, “Equity Mechanisms” (mean = 3.88), and Goal 5, “Accountability and Monitoring” (mean = 3.44). Experts may view these as cross-cutting functions that depend on robust financing and leadership rather than standalone interventions [32].

Lower-priority goals, Goal 2, “WHO Regional Reform” (mean = 5.40), and Goal 6, “Health and Non-Health Integration” (mean = 5.24), likely reflect concerns about feasibility, complexity, and limited short-term impact. These patterns mirror critiques of regional structures post-COVID-19, including coordination challenges and authority fragmentation. Goal 7, “Legal and Policy Frameworks” (mean = 4.36), elicited mixed views: while some emphasized the need for enforceability, others questioned the political viability of legal reform in a multilateral setting.

Overall, expert preferences suggest a strategic sequencing logic consistent with theories of path dependency and institutional development [32]: foundational reforms in financing and leadership must be secured before equity, legal, and multisectoral objectives can be effectively pursued

#### 4.2.2. Inter-Goal Correlation and Trade-Off Analysis

Analytical Approach: Spearman’s rank correlation was applied to the prioritization data to identify strategic alignments and trade-offs across goals. Unlike consensus scores, these correlations reveal whether experts tend to co-prioritize certain reforms (positive correlations) or view them as competing priorities (negative correlations), offering insight into how governance reforms are implicitly sequenced (Table 3).

Strategic Alignments and Tensions: A modest negative correlation between Goal 1 (WHO Leadership) and Goal 3 (Sustainable Financing) (r = −0.22) suggests that some experts view institutional authority and financial resilience as competing reform logics. This reflects two theories of change: one centred on top-down coordination, the other on resource mobilization as the foundation of system transformation, consistent with Weiss’s theory-based approach to complex change [33].

A stronger negative correlation between Goal 4 (Equity Mechanisms) and Goal 7 (Legal Frameworks) (r = −0.47) indicates an ideological divergence between distributive and procedural priorities. This underscores the need to embed equity considerations into legal reforms from the outset to ensure that enforcement mechanisms advance inclusive and rights-based outcomes [34].

By contrast, the positive correlation between Goal 5 (Accountability and Monitoring) and Goal 7 (Legal and Policy Frameworks) (r = 0.51) shows that many experts view legal enforceability and accountability as mutually reinforcing. This supports integrating accountability mechanisms within binding instruments to strengthen compliance and institutional credibility [35].

Goal 2 (WHO Regional Reform) showed generally weak or negative correlations, including a notable inverse relationship with Goal 3 (r = −0.43), suggesting strategic ambivalence. While decentralization may be normatively appealing, it is not widely viewed as an immediate driver of reform. Its role may require clearer articulation or later-stage sequencing.

Implications: These patterns highlight tensions that may hinder coherent implementation and point to the value of a phased reform roadmap. Foundational reforms, especially financing and legal frameworks, should precede more ideologically sensitive goals such as equity; legal and accountability domains should be aligned; and regional restructuring may be best deferred until core structural conditions are established. This approach aligns with institutional sequencing theory, which emphasizes establishing political and functional preconditions before pursuing more complex systemic transformations [32].

### 4.3. Structural Coherence and Goal Interdependencies

This section examines how the seven GGFH goals function as an integrated system using two approaches: (1) Pearson correlation analysis to assess consistency in expert scoring, and (2) functional interdependence mapping to explore causal linkages, synergies, and sequencing needs.

#### 4.3.1. Score-Based Pearson Correlation Analysis

A Pearson correlation matrix was generated from average Likert scores across the goals. High positive coefficients indicate alignment in expert judgments and suggest a shared evaluative framework.

**Figure 3:**
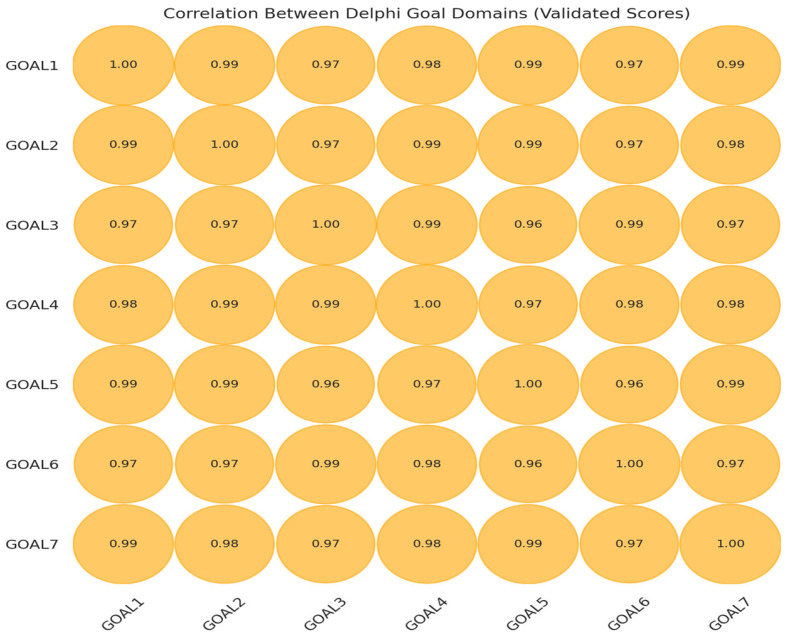
Pearson Scores Correlation Matrix, Source: Delphi Round 1 Scoring.

Key Findings: As shown in Figure 3, correlations were uniformly strong (r > 0.95), several exceeding r = 0.99. In Delphi research, such patterns typically indicate a coherent underlying logic guiding expert evaluations [13].

Highly Correlated Pairs: Near-perfect correlations (r = 0.99) were observed between:

Goal 1 (WHO Leadership) and Goal 2 (Regional Reform)

Goal 3 (Sustainable Financing) and Goal 4 (Equity Mechanisms)

Goal 5 (Accountability and Monitoring) and Goal 7 (Legal and Policy Coherence)

These associations reflect clear interdependencies. For example, the link between financing and equity suggests that equitable distribution depends on predictable funding streams, while the strong alignment between accountability and legal coherence indicates that monitoring and enforcement require robust legal instruments.

Moderate Divergence: The slightly lower correlation between Goal 5 “Accountability” and Goal 6 “Cross-Sectoral Integration” (r = 0.96) reflects the complexity of enforcing accountability across multisectoral domains, where diffuse roles and responsibilities can complicate oversight.

Overall, the consistently high correlations reinforce that the seven goals operate as components of a unified governance architecture rather than independent priorities.

From a treaty operationalization perspective, these findings support a phased yet interconnected implementation strategy, one that begins with foundational enablers such as financing, legal frameworks, and leadership, while advancing mutually reinforcing reforms in parallel. This approach aligns with institutional sequencing theory, which emphasizes establishing coherent functional foundations before pursuing more politically sensitive or operationally complex reforms [32].

#### 4.3.2. Functional Goal Interdependence Analysis

To complement the findings on consensus and prioritization, a structured interdependence matrix was introduced in Delphi Round 1 to assess how strongly each governance goal was perceived to influence the achievement of others. This approach draws on the theory of functional interdependence in institutional design, which posits that successful governance reforms often require enabling conditions, legal, financial, or normative, across distinct domains [32].

**Figure 4:**
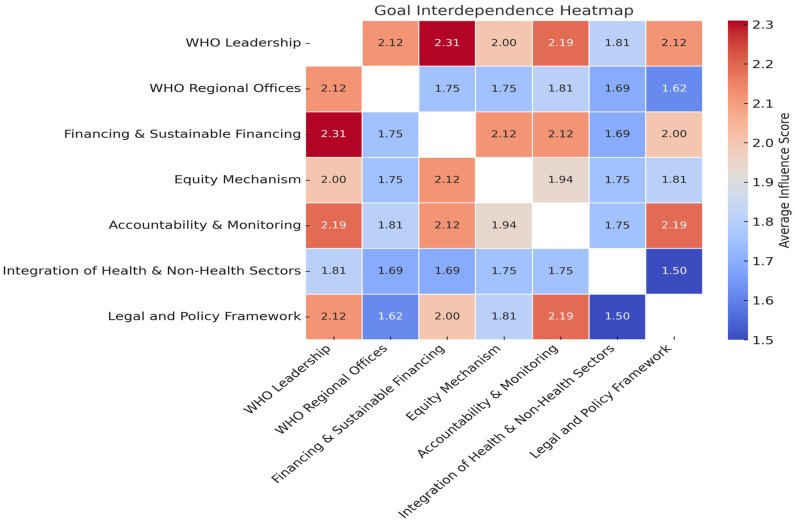
Interdependence Matrix of FGGH Goals Based on Expert Ratings (Delphi Round 1).

As shown in Figure 4, the resulting heatmap presents expert ratings of inter-goal influence on a scale from 1 (no influence) to 3 (strong influence). Higher scores indicate that the row goal is viewed as an important enabler for achieving the column goal. Lower scores suggest limited perceived influence or dependency. These patterns offer insight into the conceptual and operational linkages shaping how reforms might be bundled, sequenced, or layered in treaty implementation.

Key Insights and Strategic Implications: Financing as a Systemic Enabler: Goal 3 “Sustainable and Equitable Financing” emerged as the most structurally influential node within the interdependence matrix, particularly in relation to Goal 4 “Equity Mechanisms” (2.86), Goal 1 “WHO Leadership” (2.64), and Goal 7 “Legal and Policy Frameworks” (2.57).

These findings reinforce the role of financing as a foundational enabler. From an implementation standpoint, this supports the prioritization of Article 19 (Financing) within the WHO Pandemic Agreement for unlocking downstream institutional goals.

Goals 4 “Equity Mechanisms” and 5 “Accountability & Monitoring” exhibited reciprocal interdependence scores (2.50 in both directions), suggesting that equity cannot be meaningfully realized without robust accountability mechanisms, and that accountability, in turn, gains legitimacy through an equity-focused lens. This reinforces the need for joint operationalization of Article 9 (Equitable Access) and Article 11 (Monitoring and Evaluation) in the WHO Pandemic Agreement. Rather than treating these domains in parallel silos, they should be bundled within Phase II implementation, detailed in paragraph (3.3.2.2.) packages that focus on fairness, transparency, and legitimacy.

Legal and Policy Frameworks as Cross-Cutting Infrastructure: While Goal 7 “Legal and Policy Frameworks” did not emerge as the top influencer in any single dyadic relationship. This pattern suggests that legal and policy coherence functions as a horizontal enabler, stabilizing vertical reforms such as financing and accountability.

These findings support the strategic use of Article 13 “Legal Preparedness” in the WHO Pandemic Agreement as a scaffolding mechanism to embed legal frameworks across implementation domains, rather than addressing them in isolation.

Lagging Influence of Cross-Sectoral Integration: Goal 6 “Integration of Health and Non-Health Sectors” received the lowest interdependence scores across the matrix. This underscores the need to clearly articulate the value of intersectoral governance, and to provide support for operationalizing One Health and similar frameworks.

Recommended Phased Implementation Strategy: Drawing on institutional sequencing theory and expert-identified interdependencies, a three-phase approach is recommended for operationalizing the governance goals within the treaty framework. This model emphasizes the need to establish foundational enablers before expanding to more complex or politically sensitive reforms.

### 4.4. Expert Evaluation of Thematic Goals: Quantitative and Qualitative Insights (Supplementary Materials File S4)

This section integrates quantitative consensus measures with qualitative insights to assess expert evaluations of the proposed governance goals. Consistent with Delphi methodology, two thresholds were applied: Strict Consensus (≥75% rating ≥ 6 and SD ≤ 1.35) and Flexible Consensus (≥75% rating ≥ 5 and SD ≤ 1.5). These criteria are widely used in health policy and implementation science. When individual statements within a goal showed variation but the aggregate distribution met strict thresholds, the goal was still deemed to have achieved consensus, indicating unified support for its strategic direction despite differences in operational detail [16].

Qualitative comments were analyzed to contextualize numeric patterns, offering additional insight into areas of convergence and contention. This mixed analytical approach enhances interpretation of expert judgments by linking score distributions with the underlying technical, political, and normative reasoning.

Table 4 summarizes consensus results across all goals and statements, including medians, standard deviations, agreement levels, and whether strict or flexible thresholds were met. It also presents pre- and post-revision results for Goals 1 and 7. These goals did not initially reach strict consensus in Rounds 1and 2; however, after targeted revisions to improve clarity and operational specificity, both achieved strict consensus in Round 3. This iterative refinement process reflects the core strength of the Delphi method, structured feedback enabling convergence toward clearer, actionable priorities and improving the methodological rigour of consensus formation. (See Supplementary Materials File S5 for an expanded table of expert consensus across goals).

The following subsections present detailed findings for each thematic goal examined in the Delphi process. For each goal, both the quantitative consensus indicators and the main themes from qualitative feedback are reported. Where relevant, results are shown both before and after statement revisions.

#### 4.4.1. Goal 1: Enhancing WHO’s Leadership Role in Pandemic Governance

The Delphi process evaluated expert support for five proposed statements under this goal, with both statistical consensus measures and thematic insights assessed across Rounds 1–3.

Rounds 1&2 Summary: Across Rounds 1 and 2, three of five statements met at least one consensus threshold (Table 4).

1. Highest Support:

“Include LMICs in WHO decision-making” received the strongest endorsement (median = 7.00; SD = 0.77), with 97% ≥ 5 and 90% ≥ 6, meeting strict consensus and confirming strong support for formal LMIC representation.

2. Other Supported Items:

“WHO as Central Coordinator” (median = 7.00; SD = 0.92) and “Empower COP performance review mechanism” (median = 6.00; SD = 0.86) achieved flexible consensus (93% and 97% ≥ 5, respectively).

3. Limited Consensus:

“Create Global Health Security Unit” (SD = 1.55) and “UN–WHO Emergency Leadership” (SD = 1.59) had dispersed scores, with only 53% and 47% ≥ 6, and did not meet consensus.

4. Consensus:

The aggregate median was 6.10 (SD = 0.75), with 97% ≥ 5 and 60% ≥ 6, indicating flexible consensus for the goal overall.

5. Expert Interpretation:

Experts endorsed stronger WHO leadership but cautioned against duplication and overreach. LMIC inclusion, COP oversight, legitimacy, and role clarity emerged as the key priorities.

Round 3 Summary: Strong consensus was achieved on all five revised statements under Goal 1, with each meeting both strict and flexible criteria (Table 4).

1. Highest Support:

“Ensure equitable LMIC inclusion in WHO governance and agenda-setting” received the strongest endorsement (median = 7.00; mean = 6.73; SD = 0.59), with 100% ≥ 5 and 93% ≥ 6. Experts strongly favoured formal, substantive participation of LMICs and regional bodies (e.g., African Union, ASEAN) to strengthen legitimacy and equity.

2. Other Highly Supported Items:

“Integrate WHO existing bodies to strengthen its coordinating role” also received very high approval (median = 7.00; mean = 6.47; SD = 0.74), with 100% ≥ 5 and 87% ≥ 6, reflecting support for consolidating WHE, SCHEPPR, and IOAC to improve coherence.

“Support COP to monitor states; WHO accountability stays with WHA” (median = 6.00; mean = 6.20; SD = 1.26) met both thresholds (93% ≥ 5; 93% ≥ 6), although the higher SD suggests some concern regarding role delineation.

3. Moderate Agreement with Some Variability:

“Enhance the WHO Health Emergencies Programme; avoid parallel structures” achieved consensus (median = 6.00; mean = 6.27; SD = 0.80) with 100% ≥ 5 and 80% ≥ 6.

Similarly, “Establish a UN–WHO leadership authority using existing platforms” (median = 6.00; mean = 5.93; SD = 1.03) met criteria (93% ≥ 5; 80% ≥ 6) but drew more varied responses.

4. Consensus:

The aggregate results (median = 6.40; mean = 6.32; SD = 0.58; 100% ≥ 5; 87% ≥ 6) confirm strong and stable agreement with the refined goal.

5. Expert Interpretation:

Comments emphasized consolidating existing WHO structures rather than creating new ones, citing risks of duplication and fragmentation. Strengthening WHE, SCHEPPR, and IOAC, with clear mandates, was preferred over institutional expansion. Equity, particularly LMIC co-design, was repeatedly prioritized. Experts stressed the need for clear delineation between WHA and COP oversight to preserve constitutional coherence. They also highlighted feasibility concerns, cautioning against over-bureaucratisation and underscoring the importance of agility, transparency, and resource alignment.

Overall: Across all three rounds, expert support for reinforcing WHO’s leadership role remained consistently strong. The dominant view favoured reforms grounded in consolidation, equity, clarity of authority, and practical feasibility, rather than expanded institutional complexity

#### 4.4.2. Goal 2: Redefining the Role of WHO Regional Offices

Table 4 summarizes the findings. All five statements met both strict and flexible consensus thresholds, reflecting strong support despite divergent views on balancing regional empowerment with accountability.

1. Highest Support:

“Regional Offices as Support Hubs” received the strongest endorsement (median = 6.50; SD = 0.67), with 100% ≥ 5 and 90% ≥ 6, indicating exceptional agreement.

2. Other Strongly Supported Items:

“Strengthen Regional Competencies” (median = 7.00; SD = 0.96) and “Establish National Focal Points” (median = 7.00; SD = 1.06) were highly rated, with 97% and 87% ≥ 5, respectively.

“Allocating Direct Financing” (median = 6.50; SD = 1.21) also achieved strong support, though with slightly more variation over financial devolution.

3. Moderately Varied Views:

“Grant Regional Autonomy” (median = 7.00; SD = 1.04) met consensus but had the lowest strict-consensus score (77%), reflecting caution regarding autonomy without strong safeguards.

4. Consensus:

The overall median was 6.40 (SD = 0.61), with 97% ≥ 5 and 80% ≥ 6, confirming both strict and flexible consensus and demonstrating broad support for regional strengthening.

5. Expert Interpretation:

Experts endorsed enhanced regional roles and National Pandemic Preparedness Focal Points, noting that regional hubs are well placed to deliver context-specific and rapid support. Concerns, however, centred on risks of fragmentation, duplication, and corruption if autonomy is not paired with clear mandates, accountability mechanisms, and coordinated oversight. Direct financing was viewed as essential for agility but requiring strong fiduciary controls and equitable allocation. Some warned against excessive decentralization, while others proposed complementary innovations such as regional think tanks. Overall, experts favoured collaborative governance between the WHO headquarters, regional offices, and national authorities.

#### 4.4.3. Goal 3: Securing Sustainable and Equitable Financing

Table 4 summarizes the findings. Two of four statements met both strict and flexible consensus criteria.

1. Highest Support:

“Priority for Vulnerable Systems” received the strongest endorsement (median = 7; SD = 1.17), with 97% ≥ 5 and 93% ≥ 6, and was the only item to meet both strict and flexible consensus individually.

2. Other Highly Rated Items:

“Independent Review Board” (median = 6.37; SD = 1.56) gained strong support (93% ≥ 5; 87% ≥ 6) and met the flexible consensus threshold, though score variability prevented strict consensus.

3. Slightly Varied Support:

“Transparent Pandemic Preparedness Fund” (median = 7; SD = 1.68) and “Sustainable Financing via Multiple Sources” (median = 6; SD = 1.52) were endorsed (87% and 80% ≥ 5/6, respectively) but did not meet either consensus threshold due to more diverse expert perspectives.

4. Consensus:

At the composite level, Goal 3 achieved both strict and flexible consensus (median = 6.50; SD = 1.18; 88% ≥ 5; 78% ≥ 6), indicating broad agreement on the centrality of sustainable, equitable, and accountable financing.

5. Expert Insights:

Experts strongly supported establishing a Global Pandemic Preparedness Fund with predictable, diversified, and equity-oriented financing, emphasizing priority for fragile health systems. Concerns included the risk of politicizing vulnerability, potential inefficiencies from new oversight bodies, and duplication with existing mechanisms. Some advocated levies on high-risk industries but noted political and technical barriers. Additional issues included governance clarity, accountability during non-crisis periods, and safeguarding national investments.

Despite these variations, experts agreed that sustained financing, equitable allocation, and robust oversight are foundational to effective pandemic preparedness.

#### 4.4.4. Goal 4: Establishing Equity Monitoring Mechanisms

Table 4 summarizes the results for this goal. Of the three statements assessed, two met both strict and flexible consensus criteria.

1. Highest Support

“Equity Indicators” received the strongest endorsement (mean = 6.33; median = 7.00; SD = 1.06), with 93% ≥ 5 and 80% ≥ 6, satisfying both consensus thresholds.

2. Other Highly Rated Items

“Priority to Vulnerable Populations” (mean = 6.24; median = 6.00; SD = 0.99) was rated 90% ≥ 5 and 83% ≥ 6, also meeting strict and flexible consensus.

3. Slightly Varied Support

“Public Equity Dashboard” was positively evaluated (mean = 6.14; median = 7.00) but showed higher dispersion (SD = 1.60). Although 87% ≥ 5 and 83% ≥ 6, the variability prevented it from meeting consensus threshold.

4. Consensus

At the composite level, Goal 4 achieved both strict and flexible consensus (median = 6.33; SD = 1.03; 90% ≥ 5; 83% ≥ 6), indicating broad support for embedding equity metrics and prioritizing vulnerable populations within preparedness frameworks.

5. Expert Insights

Experts emphasized that equity must be systematically integrated into all phases of pandemic governance, with strong indicators and prioritization of under-resourced populations. While equity tools were widely supported, some questioned the feasibility and enforceability of a public dashboard. Respondents stressed that equity should be operationalised, embedded in funding, decision-making, and oversight, ensuring meaningful representation of LMICs and marginalized groups to enhance legitimacy and accountability.

#### 4.4.5. Goal 5: Adopting Accountability and Monitoring Platforms

Table 4 summarizes the findings for this goal. Of the four statements assessed, two achieved both strict and flexible consensus, and two met flexible consensus only.

1. Highest Support

The Real-Time Global Disease Surveillance Dashboard received the strongest endorsement (mean = 7.00; median = 7.00; SD = 0.86), with 97% ≥ 5 and 83% ≥ 6, confirming clear support for real-time monitoring as essential for early warning.

2. Other Strongly Supported Items

Independent Peer Review also achieved strong consensus (mean = 7.00; SD = 1.17; 90% ≥ 5, 83% ≥ 6), indicating broad endorsement of peer-based oversight for transparency and continual improvement.

3. Slightly Varied Support

Preparedness Scorecards (mean = 6.00; SD = 1.39) met flexible but not strict consensus (87% ≥ 5, 70% ≥ 6), reflecting support with less uniformity.

Mandatory publication of preparedness audits similarly met only flexible consensus (mean = 6.00; SD = 1.30; 80% ≥ 5, 73% ≥ 6), suggesting caution about enforceability and feasibility.

4. Consensus

Goal 5 achieved both strict and flexible consensus overall (median = 6.50; SD = 1.18; 88% ≥ 5, 78% ≥ 6), reflecting strong expert support for accountability mechanisms.

5. Expert Insights

Experts strongly favoured independent platforms, particularly peer reviews and real-time dashboards, as credible mechanisms to enhance trust and transparency. Many stressed the importance of regional contextualisation and independence (e.g., Africa CDC, ASEAN).

Concerns focused on bureaucratic burden, political resistance, and over-centralisation; several advocated strengthening existing tools such as JEE and SPAR. Experts emphasized that accountability mechanisms should promote learning and system improvement, not merely compliance, and noted that tools like scorecards or mandatory audits may be less effective without analytical capacity, political support, and enforcement mechanisms. Overall, transparent and well-governed accountability structures were viewed as indispensable for pandemic preparedness.

#### 4.4.6. Goal 6: Integrating Health and Non-Health Sectors

All three statements met the Flexible Consensus Criterion, and one met the Strict Criterion, indicating moderate to strong agreement, particularly regarding climate–health integration.

1. Highest Support

“Incorporate Climate Health Risk” received the strongest endorsement (mean = 6.40; median = 7.00; SD = 1.30), with 93% ≥ 5 and 93% ≥ 6, meeting both strict and flexible consensus thresholds.

2. Other Highly Rated Items

“Health in All Policies” achieved a mean of 5.87 (median = 6.00; SD = 1.38), with 87% ≥ 5 and 77% ≥ 6, meeting flexible but not strict consensus due to greater score variability.

3. Slightly Varied Support

“Partnership with Non-Health Sectors” showed broader dispersion (mean = 6.03; median = 7.00; SD = 1.50). Although 87% ≥ 5, only 70% ≥ 6, preventing strict consensus but meeting the flexible threshold.

4. Consensus

Goal 6 achieved both strict and flexible consensus at the composite level (median = 6.50; SD = 1.24; 90% ≥ 5; 77% ≥ 6), demonstrating broad albeit uneven support for whole-of-government and whole-of-society approaches.

5. Expert Comments

Experts strongly endorsed integrating pandemic prevention with broader determinants of health, especially through cross-sectoral governance, Health in All Policies (HiAP), and climate-risk frameworks. HiAP, defined as systematically incorporating health into decision-making across sectors such as environment, economy, and education, was viewed as essential for addressing upstream risks, including zoonotic spillover and climate-driven health threats [36].

Concerns centred on bureaucratic complexity, unclear mandates, and implementation challenges within current global governance structures. Some noted that mechanisms like the Quadripartite (FAO, UNEP, WHO, WOAH) already address One Health priorities, questioning the added value of new structures. Others argued that meaningful cross-sectoral integration requires institutional reform and political commitment to avoid remaining aspirational.

#### 4.4.7. Goal 7: Enforcing Legal and Policy Framework

Rounds 1 and 2 Summary: Table 4 summarizes the findings for Goal 7. Of the four statements assessed, two met the strict consensus criterion.

1. Highest Support

“Legal Assistance for LMICs/LDCs” received the strongest endorsement (median = 7; SD = 0.93), with 93% ≥ 5 and 83% ≥ 6, meeting both strict and flexible consensus thresholds. Experts highlighted that legal and technical capacity-building is essential for treaty compliance and equitable participation in global health law.

2. Other Highly Rated Items

“Treaty Compliance Review” (median = 6; SD = 1.01) also met strict and flexible consensus, with 90% ≥ 5 and 77% ≥ 6, reflecting broad support for independent mechanisms to strengthen accountability and legitimacy.

3. Slightly Varied Support

“Binding Equity/Legal Standards” reached flexible consensus only (median = 6; SD = 1.20; 80% ≥ 5, 63% ≥ 6). While experts supported binding equity norms, several noted political and enforcement constraints.

“IHR Revision” showed the widest variation (median = 6; SD = 1.51); although 83% ≥ 5, only 63% ≥ 6, and it met neither consensus criterion—indicating cautious support given ongoing debates about revising the IHR alongside a new treaty.

4. Consensus

At the composite level, Goal 7 achieved flexible consensus (median = 6.00; mean = 5.93; SD = 0.84; 90% ≥ 5, 60% ≥ 6). While strict consensus was not met, results show substantial agreement on the importance of legal accountability and support mechanisms.

5. Expert Insights

Experts emphasized the centrality of legal tools for equitable and credible pandemic governance, particularly compliance mechanisms and capacity-building for LMICs. However, they cautioned that binding obligations must be accompanied by financial and technical support to avoid overburdening states.

Views on revising the IHR were mixed: some saw expanded legal powers as necessary for enforceability, while others warned against “legal fatigue” and prioritized implementation of existing amendments. Broader concerns included political feasibility, operational complexity, and state resistance. Many experts underscored the need for cooperative; well-supported legal frameworks embedded within inclusive and regionally coordinated governance structures

Round 3 Summary: All four revised statements met both strict and flexible consensus thresholds, demonstrating strong expert support for reinforcing legal and policy mechanisms within the WHO Pandemic Agreement.

1. Highest Support

“Support Implementation of IHR (2005) Amendments” received the strongest endorsement (median = 7.00; mean = 6.60; SD = 0.63), with 100% ≥ 5 and 87% ≥ 6, confirming strict consensus and signalling clear agreement that operationalising the revised IHR is a legal priority.

2. Other Highly Rated Items

“Binding Equity Obligations (CBDR Principle)” also received strong support (median = 7.00; mean = 6.40; SD = 0.74), with 100% ≥ 5 and 87% ≥ 6, reflecting broad endorsement for embedding equity through differentiated legal commitments.

“Sustained Legal and Technical Assistance for LMICs/LDCs” met strict consensus (median = 6.00; mean = 6.27; SD = 0.88), with 100% ≥ 5 and 80% ≥ 6, underscoring the importance of long-term capacity-building.

“Cooperative Treaty Compliance Review” similarly met both thresholds (median = 6.00; mean = 6.20; SD = 0.68; 100% ≥ 5; 80% ≥ 6), indicating support for a collaborative rather than punitive compliance model.

3. Consensus

Across items, the mean score was 6.37 (median = 6.20), with 100% ≥ 5 and 84% ≥ 6, confirming strong, consistent expert alignment on legal accountability, equity, and capacity support.

4. Expert Insights and Interpretation

Experts prioritized implementation of the 2024 IHR amendments as the core legal foundation for pandemic preparedness, favouring support-based rather than coercive enforcement. Equity remained central, with strong endorsement of the CBDR principle to ensure differentiated obligations, paired with mechanisms for progressive realization and mutual accountability.

Sustained technical, legal, and financial assistance for LMICs/LDCs was frequently emphasized as essential to prevent inequitable burdens. Cooperative compliance reviews, building on tools such as JEE and SPAR, were preferred over new enforcement bodies, reflecting a desire for trust-based, familiar mechanisms.

While binding norms were seen as important for fairness and legitimacy, concerns about political feasibility, sovereignty sensitivities, and rising populism tempered expectations for strong enforcement. Overall, experts favoured a pragmatic legal architecture balancing ambition with operational and political realities.

#### 4.4.8. Goal 8: Evaluating the GGFH’s Coverage of Governance and Equity Gaps

Experts expressed strong support for the overarching principles of the proposed Global Governance for Health Framework (GGFH), particularly its emphasis on inclusivity, sovereignty, and coherence.

1. Balances Global and National Roles

This principle received the strongest endorsement (median = 7.00; 97% ≥ 6), reflecting clear agreement that effective governance must balance international coordination with national sovereignty.

2. Ensures LMIC Representation

Also highly rated (median = 7.00; 93% ≥ 6), indicating broad consensus on the need for meaningful and equitable participation of LMICs in global decision-making.

3. Addresses Governance and Equity Gaps

Experts showed confidence in the framework’s ability to reduce institutional and fairness deficits (median = 6.00; 77% ≥ 6), signalling support for its corrective emphasis despite persistent global disparities.

4. Presents a Coherent Strategic Framework

This item met the consensus threshold (median = 6.00; 77% ≥ 6) but received comparatively lower ratings, suggesting some reservations about practical implementation within politically complex environments.

**Figure 5:**
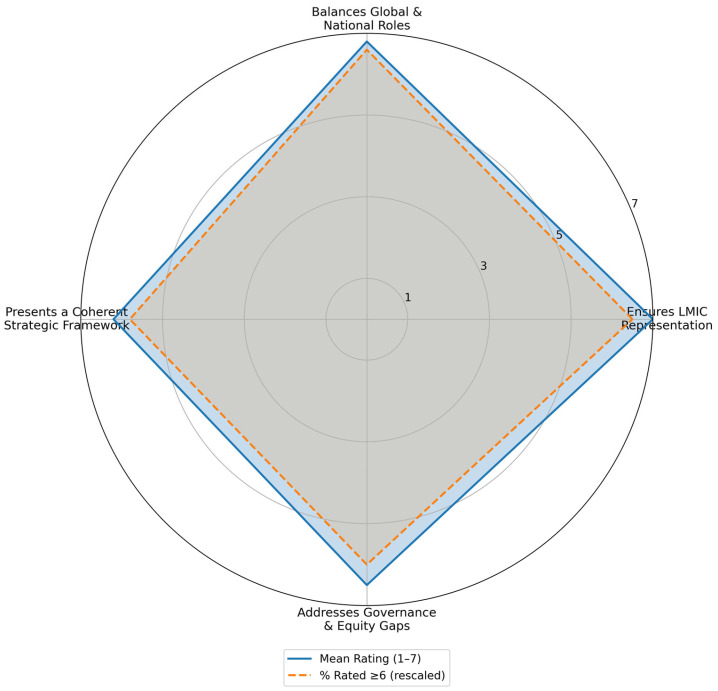
Rating of the Overarching Statements.

Figure 5 provides a visual summary of expert ratings across the four GGFH principles, illustrating areas of strong alignment and moderate variation.

## 5. Discussion

The COVID-19 pandemic revealed critical vulnerabilities in the global health governance (GHG) system, highlighting fragmentation, unequal power dynamics, and insufficient coordination across national and institutional boundaries. As the crisis unfolded, multiple actors, states, international organizations, and non-state institutions acted within their domains, often without synergy.

Global health governance is understood as the use of formal and informal institutions, rules, and processes to collectively address cross-border health challenges. However, governance performance is shaped not only by actor decisions and capacities, but also by the structure, dynamics, and regulatory frameworks within which they operate. To address these structural and functional deficits, this study proposes a Framework for Global Governance for Health (GGFH), a model that seeks to reshape how authority, accountability, and resources are distributed across the global health landscape.

### 5.1. The Proposed Global Governance for Health Framework (GGFH)

The proposed Global Governance for Health Framework (GGFH) is a multidimensional model developed to address the governance failures revealed by the COVID-19 pandemic. Validated through a Delphi process with interdisciplinary experts, it builds on the WHO Pandemic Agreement of 2025. Central to the GGFH is its response to a long-standing governance gap: the lack of cross-sectoral integration linking health with broader determinants such as environment, trade, and finance.

#### 5.1.1. Theoretical Foundation

The conceptual foundation of the framework draws from several interrelated governance theories that explain how authority, collaboration, and accountability can be organized across institutional boundaries to achieve collective goals.

1. Intersectoral Governance Theory

Intersectoral governance theory explains how distinct policy domains, such as health, environment, finance, education, and labour, coordinate strategies, align resources, and share institutional arrangements to address complex, cross-cutting challenges [4]. The GGFH translates this theory into action through mechanisms such as Health in All Policies (HiAP) platforms; Cross-sectoral coordination bodies; Integrated financing tools; and Equity dashboards tracking distributional impacts across sectors.

2. Network Governance Theory

Network governance theory explains how governance in complex and interdependent systems emerges through interconnected networks of public, private, and civil society actors, rather than through hierarchical command-and-control structures. It recognizes the existence of multiple, overlapping centres of decision-making that must coordinate across thematic, institutional, and geographic boundaries [4,22]. The GGFH applies this model by creating decentralized yet coordinated governance networks such as regional pandemic hubs, independent peer review boards, and multi-stakeholder coordination mechanisms that link the WHO, UN agencies, regional bodies, civil society, and private-sector partners.

3. Multilevel and Polycentric Governance

While intersectoral and network governance theories capture the horizontal dimensions of collaboration, multilevel governance addresses vertical coordination across scales, global, regional, national, and local. It describes how authority is dispersed across multiple, overlapping jurisdictions and how decision-making is shared among actors operating at different levels of governance [22]. This framework is especially relevant to the Global Governance Framework for Health (GGFH), which assigns distinct but interconnected roles to the WHO headquarters, regional offices, and national focal points, reflecting the need for coordinated yet context-sensitive action.

Complementing this, polycentric governance theory conceptualizes governance systems as composed of multiple, semi-autonomous centres of authority that interact and cooperate through mutual adjustment, redundancy, and shared rules [4]. In the GGFH, this is reflected in the empowerment of regional WHO bodies and national focal points that operate independently but remain aligned through globally coordinated mechanisms.

4. Good Governance Principles

Normative principles such as transparency, accountability, participation, equity, and the rule of law form the ethical and institutional foundation of the Global Governance Framework for Health (GGFH). These principles, central to the theory of good governance, ensure that governance processes are not only effective but also legitimate, fair, and inclusive [24]. In the GGFH, these values are operationalized through, including equity dashboards, independent monitoring systems, legally binding provisions, and inclusive representation of low- and middle-income countries (LMICs).

#### 5.1.2. Objectives

The GGFH framework is designed to strengthen the governance capacities that enable countries to reduce health harms, mitigate societal disruption, and respond more effectively to future pandemics. Although the objectives focus on governance arrangements rather than public health outcomes themselves, they constitute the institutional and systemic enablers through which improved health and societal results are ultimately achieved. Accordingly, the framework pursues seven interdependent objectives:

1. Enhance the WHO’s leadership and coordination role so that global responses are timely, coherent, and supported by clear lines of authority during health emergencies.
2. Strengthen the functions of WHO Regional Offices to improve regional-level preparedness, coordination, and operational support for Member States. This objective also includes a dedicated target supporting countries, particularly low- and middle-income countries, to build more resilient health systems capable of withstanding future shocks.
3. Establish sustainable and equitable financing mechanisms that ensure all countries, particularly low- and middle-income countries, have access to predictable and timely resources for preparedness and response.
4. Promote equity in access to pandemic countermeasures by advancing mechanisms for fair distribution, benefit sharing, and support to vulnerable populations.
5. Improve systems for accountability and monitoring to ensure transparency, compliance with international obligations, and continuous learning across countries.
6. Integrate health with non-health sectors, including finance, trade, environment, and security, so that multisectoral drivers of pandemic risk are managed through coherent policies.
7. Strengthen legal and policy frameworks to reinforce countries’ obligations, clarify responsibilities, and support harmonized implementation of international agreements.

Together, these seven objectives articulate the governance capacities required to build resilient systems. By enabling coherent leadership, equitable financing, multisectoral coordination, and strong legal foundations, they provide the institutional conditions through which countries can ultimately reduce mortality, protect societies, and minimize the disruptive impact of future pandemics.

**Figure 6:**
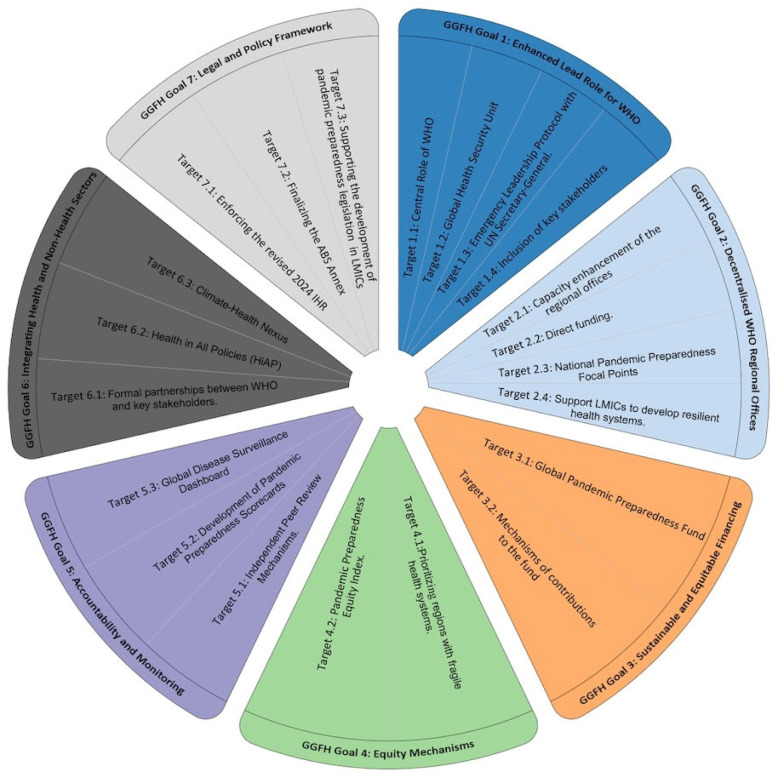
GGFH Goals and Targets.

These overarching objectives are operationalized through seven thematic goals, such as equity, accountability, and institutional leadership, each linked to specific, actionable targets. The diagram, in Figure 6, illustrates how these goals collectively structure the Global Governance for Health Framework (GGFH) into a coherent set of reform mechanisms.

#### 5.1.3. Key Components of the Framework

The GGFH framework identifies the WHO as the central authority for pandemic preparedness, supported by a Global Health Security Unit and a Global Pandemic Coordination Board. This Board, activated during emergencies, includes stakeholders such as LMIC representatives, NGOs, and global financial institutions, ensuring inclusive and coordinated decision-making.

A central innovation is the Global Pandemic Preparedness Fund, designed to finance vaccine R&D, medical stockpiles, and health system strengthening in low-resource regions. The fund would be sourced from national contributions, philanthropic entities, and levies on high-risk industries. Allocation is guided by an Equity-Based Index, a transparent, data-driven tool that prioritizes regions based on need, vulnerability, and capacity.

To promote regional ownership, WHO regional offices would receive direct resources and implementation authority to develop locally tailored preparedness plans. National Pandemic Preparedness Focal Points would coordinate country-level actions and liaise with regional hubs.

Accountability mechanisms include (1) Independent peer reviews against standardized benchmarks, (2) A Global Disease Surveillance Dashboard for real-time data monitoring, and (3) Public Pandemic Preparedness Scorecards that track progress on health system resilience and equity.

To ensure intersectoral coherence, the framework calls for Health in All policies (HIA) for non-health sectors like trade, urban planning, and environmental domains. Formal partnerships with agencies such as FAO (FAO: Food and Agriculture Organization of the United Nations) and UNEP (UNEP: United Nations Environment Programme) would facilitate this integration.

Operational tools include the deployment of rapid response teams, regional medical stockpiles, and knowledge-sharing platforms to ensure real-time coordination and equitable access during crises.

Legal reform is another pillar, including enforcement of the International Health Regulations (IHR), as well as technical assistance to help countries strengthen national pandemic legislation. A phased implementation approach, beginning with political endorsement, followed by financial and governance mechanisms, is proposed to ensure sustainability and scale.

**Figure 7:**
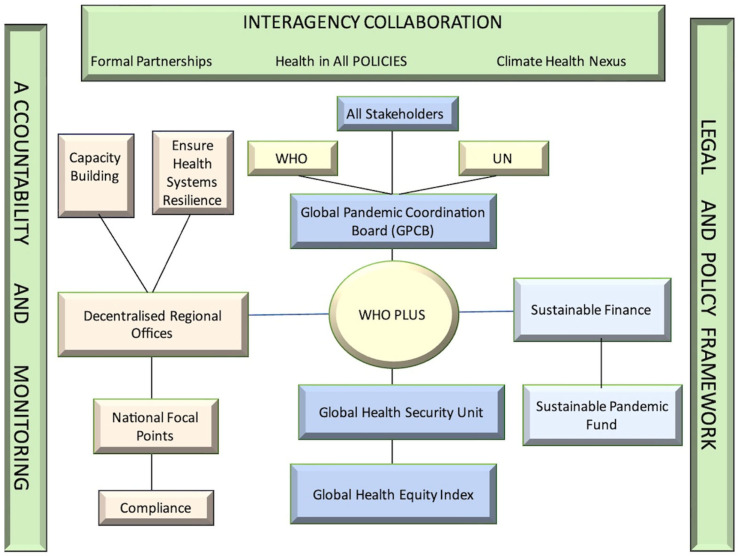
Design of the GGFH Framework.

Figure 7 provides a visual consolidation of the GGFH framework’s institutional architecture. It maps the relationships among core components, such as WHO leadership, regional implementation, intersectoral collaboration, and accountability mechanisms, highlighting how the framework operationalizes its key principles across legal, financial, and governance domains. This schematic complements the narrative overview by clarifying structural linkages and functional roles within the proposed global health governance model.

#### 5.1.4. Delphi Analysis

The framework envisions a collaborative, adaptive, and inclusive system that aligns governance processes, mobilizes diverse actors, and fosters equitable global health outcomes. It offers a structured, pragmatic approach to strengthening health governance in an increasingly interconnected world.

Its objectives include reinforcing WHO leadership, enabling sustainable financing, decentralizing authority to regional levels, enhancing multisectoral coordination, fostering inclusive decision-making, and improving pandemic preparedness through transparent, accountable, and equity-driven mechanisms.

In this context, the current study tested the Global Governance Framework for Health (GGFH) through a three-round Delphi process with global health experts. The framework aims to assess and guide reforms across seven thematic goals by aligning expert consensus on governance priorities, interdependencies, and implementation strategies.

The Delphi process validated the proposed Global Governance Framework for Health (GGHF) as both conceptually robust and operationally relevant. All seven thematic goals achieved strict consensus by the conclusion of the third Delphi round, reflecting high convergence among experts on the framework’s coherence, relevance, and alignment with the WHO Pandemic Agreement (2025). This resounding consensus followed an iterative process in which expert commentary from Rounds 1 and 2 informed substantive revisions to statements on Goal 1” WHO leadership”, and Goal 7 “legal frameworks”. These revised statements, tested in Round 3, subsequently achieved strict consensus, demonstrating the value of structured feedback loops in refining complex governance proposals.

Achieving final consensus across all goals represents a key methodological and policy milestone. The earlier divergence around Goals 1 and 7 stemmed from long-standing tensions over institutional centralization, legal enforceability, and the complexity of legal pluralism in global health governance. Some experts expressed caution regarding the feasibility of centralizing authority within the WHO or revising international legal instruments amid geopolitical tensions. However, after clarifications were introduced, such as emphasizing integration with existing WHO structures, time-bound emergency authorities, and capacity-building for LMICs, support solidified. These shifts indicate that expert resistance stemmed not from rejection of the governance goals per se, but from concerns over institutional realism and political tractability.

This shift in consensus also reveals a critical epistemic insight: experts are more likely to support ambitious governance proposals when those proposals are paired with clear operational pathways and safeguards. This is particularly evident in the revised legal statements, which coupled calls for enforceability with provisions for technical assistance and equitable burden-sharing. Similarly, previously contested financing statements gained support once reframed to emphasize inclusivity, LMIC representation, and accountability via independent oversight bodies.

The findings also underscore a high degree of internal coherence within the FGGH. Quantitative correlation analyses reveal strong alignment across governance goals, suggesting that experts conceptualize these domains not as siloed reforms but as components of a systemic architecture. Financing, legal frameworks, and leadership were perceived as foundational enablers, while equity, accountability, and integration were seen as dependent yet essential pillars. This logic is reinforced by the goal interdependence matrix, which confirms functional complementarities, especially between financing and equity, and between legal reform and accountability mechanisms.

Taken together, the study’s Delphi outcomes substantiate the GGFH as a theoretically grounded and empirically validated governance model. It addresses key structural limitations of the WHO Pandemic Agreement, including its vague compliance mechanisms, underdeveloped regional architecture, and limited financing detail. By aligning expert judgement with policy design, the framework offers a scalable roadmap for treaty operationalization and institutional reform.

Future research should focus on piloting the framework in select countries or regions, enabling adjustments based on operational realities, and involving diverse stakeholders, including civil society and frontline health workers, to capture perspectives not fully represented in the initial panels.

#### 5.1.5. Implications for Policy and Practice

Building on the phased sequence identified in Section 4.3.2. policymakers should adopt a modular implementation strategy in which foundational legal, leadership and financing reforms are prioritized first, creating momentum and capacity for later equity, accountability and cross-sectoral reforms.

1. Sequence Reform Around Structural Enablers

The prioritization of leadership, legal authority, and financing indicates that governance reform must begin by strengthening institutional scaffolding. Establishing a permanent global preparedness fund with equitable representation, legally binding compliance mechanisms, and a clarified WHO coordination mandate emerged as essential prerequisites for advancing other goals.

2. Equity Requires Institutional Infrastructure

Although equity was consistently endorsed as a normative priority, the Delphi process revealed that experts viewed it as structurally dependent on prior legal, financial, and accountability arrangements. Rather than treating equity as an aspirational principle, it must be embedded in operational mechanisms, such as allocation formulas, legal protections, and inclusive leadership models.

3. Decentralization Must Be Matched with Capacity Building, and Oversight

Support for enhanced autonomy of regional WHO offices was conditional on the presence of strong fiduciary controls, technical competencies, and vertical coordination with national focal points. This aligns with principles of multilevel and polycentric governance, which emphasize contextual flexibility within an overarching framework of coherence and accountability.

4. Legal Reforms Must Be Paired with Support for LMICs

Endorsement of legal enforceability increased significantly when paired with proposals for differentiated obligations and technical assistance for LMICs. Experts emphasized that treaty compliance must be equitable, reflecting variations in national legal infrastructure and capacity. Legal pluralism must be addressed through co-development, not imposition.

5. Institutional Innovation for Intersectoral Integration

While cross-sectoral coordination received lower initial priority, consensus affirmed its critical role in pandemic prevention. Experts called for the creation of dedicated multisectoral bodies or coordination platforms that institutionalize the One Health and Health in All Policies approaches, linking public health to trade, climate, and urban policy domains.

6. Adopt a Phased, Modular Implementation Strategy

A staged implementation process, beginning with politically and technically feasible reforms, was widely favoured. By aligning reform sequencing with expert-assessed goal dependencies, early progress in legal and financial domains can generate political momentum and stakeholder confidence for more complex institutional transformations.

7. Institutionalize Feedback Loops and Performance Monitoring

Experts highlighted the need for transparent, adaptive accountability mechanisms. These include preparedness dashboards, independent peer review cycles, and publicly accessible progress scorecards. Such tools not only enforce compliance but also support institutional learning and course correction in real time.

Synthesis: The Delphi outcomes affirm the GGFH as both conceptually coherent and practically viable. Importantly, the findings signal a shift from fragmented, market-driven global health responses toward a governance model grounded in public goods, enforceable rules, and shared responsibility. For policymakers and treaty negotiators, the GGFH offers an empirically validated and theoretically informed roadmap toward a more resilient, inclusive, and coordinated global health system.

#### 5.1.6. Limitations

While the Delphi method provided valuable insights into expert consensus on global health governance reform, several recognized limitations should be noted: First, panel composition, though diverse by sector and region, underrepresented certain key groups, specifically frontline health workers and policymakers from vulnerable areas, which may have narrowed the range of perspectives reflected in the findings.

Despite the structured selection process, participation may still reflect the voluntary interest of experts familiar with global governance issues. This limitation is inherent to purposive Delphi sampling but was mitigated through diverse disciplinary and regional representation.

Second, reliance on iterative, written responses can restrict the richness of feedback and may discourage deep engagement from experts with time or language barriers. Although efforts were made to maximize accessibility, some data may lack desired nuance or completeness.

Third, the phrasing and structure of Delphi statements may have shaped response interpretation. Despite rigorous pilot testing for clarity, conceptual ambiguity remained possible given the complexity of governance constructs.

Finally, while the framework achieved conceptual validation, it was not tested for feasibility or adaptability in real-world institutional and geopolitical contexts. Future research should focus on piloting the framework in select countries or regions, enabling adjustments based on operational realities, and involving diverse stakeholders, including civil society and frontline health workers, to capture perspectives not fully represented in the initial panels.

## 6. Conclusions

The Global Governance for Health Framework (GGFH) offers a structured response to the persistent gaps in global health governance revealed by the COVID-19 pandemic. Grounded in established governance theories and validated through expert consensus, it presents a pragmatic and adaptable model for operationalizing reforms under the WHO Pandemic Agreement. By emphasizing equity, legal enforceability, sustainable financing, and inclusive decision-making, the GGFH provides a coherent pathway toward a more resilient, accountable, and coordinated global health system, one capable of meeting current challenges and anticipating future threats.

## 7. Summary

This study presented the development and validation of the proposed Global Governance Framework for Health (GGFH), grounded in multilevel governance, collective action, meta-governance, and good governance theories. Building on insights from the COVID-19 crisis and recognizing the limitations of the WHO Pandemic Agreement, the GGFH presents a practical framework to enhance global coordination, promote equity, ensure accountability, and strengthen legal mechanisms in health governance.

Using a structured Delphi method, expert consensus was achieved across seven thematic goals of the framework, affirming its conceptual soundness and operational relevance. The analysis revealed strong interdependencies between governance functions, highlighting the need to prioritize systemic enablers such as financing, legal instruments, and leadership structures as precursors to equitable and effective pandemic preparedness.

The chapter also identified implementation considerations, including the need for phased reform, support for low- and middle-income countries, and mechanisms to ensure regional and sectoral alignment. While the study contributes a validated roadmap for reform, its limitations underscore the need for ongoing empirical testing and broad stakeholder engagement to ensure practical applicability and political feasibility.

Together, the findings underscore the potential of the GGFH framework to serve as a strategic guide for institutional reform in global health governance, offering a more integrated, inclusive, and resilient approach to managing future global health threats.

## Figures and Tables

**Table 1:** Consensus Criteria.

Consensus Criteria		
Variable	Strict	Flexible
Median	6	5
Standard Deviation	1.35	1.5
Consensus agreement	75%	75%

**Table 2:** Gender and Age Distribution.

Category	Subcategory	Frequency	Percentage
Gender	Female	16	51.6%
	Male	15	48.4%
	Total	31	100%
Age Group	30–35	3	9.6%
	45–49	10	32.3%
	50–59	12	38.7%
	60 and above	6	19.4%
	Total	31	100%

**Table 3:** Spearman Correlation Matrix Between Prioritized Goals (Source: Delphi Round 1).

	GOAL1	GOAL2	GOAL3	GOAL4	GOAL5	GOAL6	GOAL7
GOAL1	1.00	0.25	−0.22	−0.09	−0.48	−0.13	−0.47
GOAL2		1.00	−0.43	0.16	−0.17	−0.17	−0.09
GOAL3			1.00	0.32	0.06	−0.18	−0.24
GOAL4				1.00	−0.36	0.02	−0.47
GOAL5					1.00	−0.02	0.51
GOAL6						1.00	−0.25
GOAL7							1.00

**Table 4:** Expert Consensus Ratings Across Goals.

Goal	Round	No. of Statements	Aggregate Median	Aggregate SD	% ≥5	% ≥6	Strict Consensus	Flexible Consensus
Goal 1: WHO Leadership Role	R1–2	5	–	–	–	–	NO	YES
	Final	5	6.4	0.58	100%	87%	YES	YES
Goal 2: redefined Role of the WHO Regional Offices	Final	5	6.4	0.61	97%	80%	YES	YES
Goal 3: Sustainable and Equitable Financing	Final	4	6.5	1.24	90%	80%	YES	YES
Goal 4: Equity Mechanisms	Final	3	6.33	0.89	87%	77%	YES	YES
Goal 5: Accountability and Monitoring	Final	4	6.5	1.18	88%	78%	YES	YES
Goal 6: Cross-Sectoral Integration	Final	3	6.5	1.24	90%	77%	YES	YES
Goal 7: Legal and Policy Framework	R1–2	4	–	–	–	–	NO	YES
	Final	4	6.5	0.4	100%	93%	YES	YES
Goal 8: Overall Framework Validation	Final	4	6.5	0.67	97%	77%	YES	YES

## Data Availability

The original contributions presented in the study are included in the article, further inquiries can be directed to the corresponding author.

## Supplementary Materials

The following supporting information can be downloaded at: https://www.mdpi.com/article/10.3390/ijerph23010138/s1, File S1: Delphi Survey Statements Table; File S2: Table Comparing WHO Pandemic Agreement Articles Gaps vs. Delphi Statements; File S3: Updated Delphi Statements Table for Goals 1 & 7; File S4: Summary Statistical Tables; File S5: Table of Expert Consensus Ratings Across Goals.
