# Supplementary material for: Developing and Validating a Global Governance Framework for Health: A Delphi Consensus Study
Source: Int J Environ Res Public Health. 2026 Jan 22;23(1):138. doi: 10.3390/ijerph23010138 (PMC12840802; doi:10.3390/ijerph23010138)
Supplement: Supplementary file 1 [file ijerph-23-00138-s001.zip › File S1 - Delphi Survey Statements Table.pdf]

Delphi Survey Statements Table[i]

Instructions: Please rate each statement on a 1–7 Likert scale, where 1 = Strongly Disagree and 7 = Strongly Agree. Each section includes explanatory notes to clarify the policy relevance, and relation to the WHO Pandemic Agreement (2025). Comments are optional but deeply appreciated.

**1. Enhancing WHO's Leadership Role in Pandemic Governance** (*Articles 3, 22*). While Article 3 and 22 of the WHO Pandemic Agreement affirm WHO's leadership role, they lack detailed provisions on structural reforms or operational bodies needed to realize this role effectively. The proposed enhancements aim to ensure clarity, coordination authority, and equitable stakeholder inclusion.	**Score (1–7)**	**Comments**
WHO should be strengthened as the central coordinating body for global pandemic preparedness and response.		
The creation of a dedicated Global Health Security Coordination Unit within WHO will improve the organization's ability to manage future pandemics (*Not addressed in treaty*).		
A UN-WHO Emergency Leadership Authority should be established to provide binding oversight and unified command during global health crises. (Not currently addressed in treaty)		
Effective pandemic governance requires formal inclusion of multilateral institutions and low- and middle-income countries (LMICs) in WHO decision-making processes.		
The Conference of the Parties (COP) As the governing body of the WHO Pandemic Agreement, should be empowered to review the performance of all stakeholders, including the WHO, and enforce reforms where needed.		
**2. Redefined role of the WHO Regional Offices** (*Articles 15 and 17*) While Articles 15 and 17 acknowledge the importance of regional and national planning, they lack provisions for financial autonomy or local operational flexibility. The proposed refinements strengthen regional responsiveness and align governance structures with country-specific needs.	Score (1–7)	Comments
Strengthening the technical and strategic competencies of WHO regional offices will help in delivering context-specific pandemic preparedness plans.		
Allocating direct financing to WHO regional offices will enhance their operational capacity to respond rapidly to health emergencies.		
Establishing National Pandemic Preparedness Focal Points in every country will improve alignment between national strategies and global policies.		
Regional offices should serve as primary hubs for delivering targeted technical and operational support to LMICs, to strengthen national health systems and workforce capacity.		
Regional offices should be granted greater autonomy to develop preparedness strategies tailored to local contexts.		
**3. Sustainable and Equitable Financing** ***(Article 18)*** Article 18 establishes a financing framework but leaves questions about governance, equity-based allocation, and long-term sustainability. These statements propose concrete funding mechanisms and governance safeguards to ensure transparency and fair resource distribution.	Score (1–7)	Comments
A well-governed, transparent Global Pandemic Preparedness Fund, established as a permanent mechanism rather than a crisis-driven response, is important for ensuring sustainable and equitable financing for health security at all times.		
The Global Pandemic Preparedness Fund should ensure sustainable, equitable financing, supported by member states, the private sector, and levies on high-risk industries.		
Allocation criteria should explicitly prioritize countries with fragile health systems and regions most vulnerable to future pandemics.		

The fund should be overseen by an independent review board with transparent reporting and representation from LMICs.		
**4. Equity Mechanisms** *(Articles 3, 10)*<br>Despite frequent reference to equity, the treaty lacks metrics or systems for monitoring equitable outcomes. These proposals emphasize accountability through measurable equity indicators and accessible public dashboards.	Score (1–7)	Comments
Equity considerations must guide emergency response efforts, with priority given to vulnerable populations and under-resourced health systems.		
Developing a Pandemic Preparedness Equity Index can support transparent and data-informed resource allocation.		
Equity outcomes should be regularly tracked and published through a global, publicly accessible dashboard to ensure accountability.		
**5. Accountability and Monitoring** *(Article 5, 21)*<br>Although Articles 5 and 21 address compliance and reporting, they lack concrete mechanisms for external evaluation and performance comparison. The following statements propose enhanced accountability measures to strengthen transparency and global oversight**.**	Score (1–7)	Comments
Regular, independent peer reviews of national and regional pandemic preparedness capacities are important for transparency and continuous improvement.		
Publishing standardized preparedness scorecards will support comparative evaluation and public accountability.		
A real-time Global Disease Surveillance Dashboard, coordinated by WHO, will strengthen early warning systems and response readiness.		
Countries are mandated to publish preparedness audits and response assessments as part of treaty compliance and international transparency obligations.		
**6. Integrating Health and Non-Health Sectors** *(Article 5).* While the treaty adopts a One Health approach, cross-sectoral coordination mechanisms remain vague. These items stress formalized structures and integration with non-health sectors to operationalize a whole-of-government strategy.	Score (1–7)	Comments
Formal partnerships between WHO and non-health sectors (e.g., FAO, UNEP, WTO) are important to addressing upstream pandemic drivers.		
Institutionalizing a "Health in All Policies" (HiAP) approach will ensure health considerations are embedded across sectors such as trade, education, housing, and environment.		
Pandemic preparedness must incorporate climate-related health risks, to address emerging cross-sectoral health threats.		
**7. Legal and Policy Framework** ***(Related to Articles 6, 17 and IHR reform discussions)***	Score (1–7)	Comments
The International Health Regulations (IHR) must be revised to impose binding, enforceable obligations for pandemic preparedness and response.		
The effectiveness of the WHO Pandemic Agreement (2025) depends on the adoption of **binding provisions** and **equity-driven legal standards** that apply uniformly across high, middle, and low-income countries.		
WHO and relevant international bodies should provide **technical and legal assistance** to countries, particularly LMICs and LDCs,to support the drafting, revision, and enforcement of national legislation that aligns with treaty obligations.		
A **global treaty compliance review mechanism** should be created to conduct **regular legal audits** of national pandemic laws and policies, benchmarked against the IHR and the Pandemic Agreement.		
**8. General/Overarching Statements**<br>The framework aims to fill governance and equity gaps not fully resolved by the WHO Pandemic Agreement. These statements assess its comprehensiveness, inclusivity, and adaptability in the post-treaty context.	Score (1–7)	Comments

The proposed Framework for Global Governance for Health (FGGH) addresses the institutional, equity, and coordination gaps exposed during the COVID-19 response and those partially unresolved by the 2025 WHO Pandemic Agreement.		
Balancing global coordination with respect for national sovereignty is a core challenge in implementing the FGGH.		
Ensuring robust and equitable representation of LMICs in decision-making is important to building legitimacy and accountability.		
The seven goals of the FGGH offer a coherent, actionable roadmap to strengthen global pandemic preparedness.		
**9. Goal Prioritization** From your perspective, how would you prioritize the following goals based on their overall value for improving global pandemic preparedness and governance? Please consider not only their strategic importance, but also their practicality, applicability across diverse country contexts, and alignment with real-world implementation dynamics. Assign a unique rank from 1 (highest priority) to 7 (lowest priority); no two goals should share the same rank.		
Goal 1: Strengthened WHO Leadership		
Goal 2: Redefined WHO Regional Offices		
Goal 3: Sustainable and Equitable Financing		
Goal 4: Equity Mechanisms		
Goal 5: Accountability and Monitoring		
Goal 6: Integration of Health and Non-Health Sectors		
Goa**Shading and italicization are applied for visual presentation only and carry no substantive or analytical meaning.** Bold formatting indicates the primary governance objective, while non-bold text represents related sub-components within the same objective.l 7: Legal and Policy Framework		

10. Goal Interdependencies
In your view, which of the goals are most interdependent or mutually reinforcing? Please evaluate the strength of each relationship.
Instructions:
For each cell in the matrix, assign a score from 0 to 3 indicating how strongly the goal in the row influences the goal in the column:
0 = No meaningful influence; 1 = Low influence; 2 = Moderate influence; 3 = Strong influence
You may add brief notes to justify high-impact scores (2–3).
This scoring allows us to assess not only the presence of interdependencies, but also their relative strength.
Use "–" for diagonal cells where a goal would influence itself.

	**WHO Leadership**	**WHO Regional Offices**	**Financing& Sustainable Financing**	**Equity Mechanism**	**Accountability and Monitoring**	**Integration of Health and Non-Health Sectors**	**Legal and Policy Framework**
WHO Leadership							
WHO Regional Offices							
Equitable and Sustainable Financing							
Equity Mechanism							
Accountability & Monitoring							
Integration of Health and Non - Health Sectors							
Legal and Policy Framework							
