# Supplementary material for: Developing and Validating a Global Governance Framework for Health: A Delphi Consensus Study
Source: Int J Environ Res Public Health. 2026 Jan 22;23(1):138. doi: 10.3390/ijerph23010138 (PMC12840802; doi:10.3390/ijerph23010138)
Supplement: Supplementary file 1 [file ijerph-23-00138-s001.zip › File S2 - Table Comparing WHO Pandemic Agreement Articles Gaps vs. Delphi Statements .pdf]

Table Comparing WHO Pandemic Agreement Articles Gaps vs. Delphi Statements

This table organizes the 32 Delphi statements by the specific WHO Pandemic Agreement articles they correspond to. Each Delphi item addresses a gap in the article's language, implementation, or enforcement mechanism.

Article(s)	Identified Gap in WHO Pandemic Agreement	Corresponding Delphi Statement
Articles 3, 22/Goal 1	WHO leadership affirmed but lacks operational structure	WHO should be strengthened as the central coordinating body for global pandemic preparedness and response.
	Lack of structural reforms under WHO leadership	Create a Global Health Security Coordination Unit within WHO.
	No binding oversight mechanism for health emergencies	Establish a UN–WHO Emergency Leadership Authority.
	Exclusion of LMICs from decision-making bodies	Formally include LMICs in WHO decision-making processes.
	COP lacks formal performance review authority	Empower COP to review WHO and enforce reforms.
Articles 15, 17/Goal 2	Regional planning noted but lacks financial autonomy	Strengthen technical and strategic competencies of WHO regional offices.
	No provision for direct funding of regional offices	Allocate direct financing to WHO regional offices.
	No mechanism to align national and global preparedness	Establish National Pandemic Preparedness Focal Points.
	Unclear delivery role of regional offices	Regional offices as primary hubs for LMIC technical support.
	Lack of strategic autonomy for regional preparedness	Grant autonomy to regional offices for context-specific strategies.
Article 18/Goal 3	Financial mechanism lacks permanence or transparency	Create a well-governed Global Pandemic Preparedness Fund.
	Sustainability of funding not addressed	Support sustainable financing via multi-source contributions.
	No prioritization for vulnerable health systems	Prioritize funding for fragile health systems.
	No independent financial oversight body	Oversee fund with an independent review board including LMICs.
Articles 3, 10/Goal 4	Equity is stated but not operationalized	Equity must guide emergency response with priority to vulnerable groups.
	No standard equity measurement tools	Develop a Pandemic Preparedness Equity Index.
	No mechanisms to track equity outcomes	Track and publish equity outcomes through a global dashboard.
Articles 5, 21/Goal 5	No external performance evaluation mechanism	Conduct independent peer reviews of preparedness.
	Lack of standardized metrics for accountability	Publish standardized preparedness scorecards.
	Lack of real-time disease monitoring tools	Implement a Global Disease Surveillance Dashboard.
	No requirement for audit publication	Mandate countries to publish preparedness audits.
Article 5/Goal 6	One Health integration remains vague	Formalize partnerships between WHO and non-health sectors.

	No institutional mechanism for cross-sector alignment	Institutionalize Health in All Policies (HiAP).
	Climate-health linkages not addressed	Integrate climate-related health risks into preparedness.
Articles 6, 17/Goal 7	Legal obligations are broad and non-binding	Revise IHR to impose binding obligations.
	No legal equity requirements across income groups	Adopt binding equity-driven legal standards.
	Lack of support for legal compliance in LMICs	Provide legal/technical support to LMICs.
	No compliance audit mechanism	Create a treaty compliance review body.
