# Supplementary material for: Developing and Validating a Global Governance Framework for Health: A Delphi Consensus Study
Source: Int J Environ Res Public Health. 2026 Jan 22;23(1):138. doi: 10.3390/ijerph23010138 (PMC12840802; doi:10.3390/ijerph23010138)
Supplement: Supplementary file 1 [file ijerph-23-00138-s001.zip › File S3 - Updated Delphi Statements Table for Goals 1 & 7.pdf]

Updated Delphi Statements Table for Goals 1 & 7

Goal 1: Enhancing WHO's Leadership Role in Pandemic Governance (Articles 3, 22).

Original Statement	Revised Statement
WHO should be strengthened as the central coordinating body for global pandemic preparedness and response.	The World Health Organization (WHO) should be strengthened as the central coordinating body by clarifying the roles of existing bodies, such as the WHO Health Emergencies Programme (WHE), Standing Committee on Health Emergency Prevention, Preparedness and Response (SCHEPPR), and the Independent Oversight and Advisory Committee (IOAC), and integrating them into a clear coordination and accountability structure.
The creation of a dedicated Global Health Security Coordination Unit within WHO will improve the organization's ability to manage future pandemics.	A dedicated coordination mechanism for global health security should be developed within the WHO by building on and enhancing existing units like the WHO Health Emergencies Program (WHE), rather than establishing new parallel structures.
A UN–WHO Emergency Leadership Authority should be established to provide binding oversight and unified command during global health crises.	A joint United Nations–World Health Organization (UN–WHO) Emergency Leadership Authority should be developed with a clearly defined coordination mandate and time-bound crisis authority, leveraging existing platforms like the United Nations Crisis Management Team and WHO Emergency Response Framework.
Effective pandemic governance requires formal inclusion of multilateral institutions and low- and middle-income countries (LMICs) in WHO decision-making processes.	Effective pandemic governance should ensure formal and equitable representation of multilateral institutions (e.g., African Union, ASEAN) and low- and middle-income countries (LMICs) in WHO governance bodies with meaningful roles in decision-making and agenda-setting.
The Conference of the Parties (COP) as the governing body of the WHO Pandemic Agreement should be empowered to review the performance of all stakeholders, including the WHO, and enforce reforms where needed.	The Conference of the Parties (COP) should focus on monitoring state party compliance with the WHO Pandemic Agreement, while the performance review of the World Health Organization (WHO) remains under the authority of the World Health Assembly (WHA), in line with the WHO Constitution.

Goal 7: Legal and Policy Framework for Compliance and Equity (Articles 6, 17, and IHR).

Original Statement	Revised Statement
The International Health Regulations (IHR) must be revised to impose binding, enforceable obligations for pandemic preparedness and response.	The 2024 amendments to the International Health Regulations (IHR) should be implemented as the primary legal framework for pandemic preparedness and response, with a focus on supporting national compliance through technical and financial assistance.
The effectiveness of the WHO Pandemic Agreement depends on binding, equity-driven legal standards applicable across all income levels.	The WHO Pandemic Agreement should embed binding obligations based on the principle of Common but Differentiated Responsibilities (CBDR), ensuring equitable commitments tailored to national capacity.
WHO and relevant bodies should provide technical and legal assistance to support LMICs/LDCs in aligning national legislation with the treaty.	The World Health Organization (WHO), in coordination with regional institutions (e.g., African Union, ASEAN), should provide sustained technical and legal support to low- and middle-income countries (LMICs) and least developed countries (LDCs) to align national legislation with treaty obligations.

A global treaty compliance review mechanism should be created to audit and benchmark national laws.	A cooperative and transparent treaty compliance review mechanism should be established, building on existing tools such as the Joint External Evaluation (JEE), State Party Self-Assessment Annual Reporting (SPAR), and regional peer-review platforms to promote mutual accountability.
