# Supplementary material for: Developing and Validating a Global Governance Framework for Health: A Delphi Consensus Study
Source: Int J Environ Res Public Health. 2026 Jan 22;23(1):138. doi: 10.3390/ijerph23010138 (PMC12840802; doi:10.3390/ijerph23010138)
Supplement: Supplementary file 1 [file ijerph-23-00138-s001.zip › File S4 - Summary Statistical Tables.pdf]

Summary Statistical Tables

Table S1. Goal 1. Enhancing WHO's Leadership Role in Pandemic Governance (R1&2).

	WHO as Central Coordinator	Create Global Health Security Unit	UN-WHO Emergency Leadership	Include LMICs in WHO Decision Making	Empower COP Performance Review	Aggregate
Mean	6.30	5.47	5.23	6.60	6.13	5.95
Std Dv	0.92	1.55	1.59	0.77	0.86	0.75
Median	7.00	6.00	5.00	7.00	6.00	6.10
High	7	7	7	7	7	7
Low	4	1	1	4	4	3.8
Top Quartile	7	7	6	7	7	6.4
Bottom Quartile	6	4	5	6.5	6	5.5
Interquartile Range	1	3	1	0.5	1	0.9
#above or equal to 5	28	21	23	29	29	29
% above or equal to 5	93%	70%	77%	97%	97%	97%
#above or equal to 6	25	16	14	27	23	18
% above or equal to 6	83%	53%	47%	90%	77%	60%
**Strict Consensus Criterion**	**YES**	**FALSE**	**FALSE**	**YES**	**YES**	**FALSE**
**Flexible Consensus Criterion**	**YES**	**FALSE**	**FALSE**	**YES**	**YES**	**YES**

Table S2. Goal 1. Enhancing WHO's Leadership Role in Pandemic Governance (R3).

	WHO as Central Coordinator	Create Global Health Security Unit	UN-WHO Emergency Leadership	Include LMICs in WHO Decision Making	Empower COP Performance Review	Aggregate
Mean	6.47	6.27	5.93	6.73	6.20	6.32
Std Dv	0.74	0.80	1.03	0.59	1.26	0.58
Median	7.00	6.00	6.00	7.00	6.00	6.40
High	7	7	7	7	7	7
Low	5	5	3	5	2	5.2
Top Quartile	7	7	6.5	7	7	6.8
Bottom Quartile	6	6	6	7	6	6
Interquartile Range	1	1	0.5	0	1	0.8
#above or equal to 5	15	15	14	15	14	15
% above or equal to 5	100%	100%	93%	100%	93%	100%
# above or equal to 6	13	12	12	14	14	13
% above or equal to 6	87%	80%	80%	93%	93%	87%
**Strict Consensus Criterion**	**YES**	**YES**	**YES**	**YES**	**YES**	**YES**
**Flexible Consensus Criterion**	**YES**	**YES**	**YES**	**YES**	**YES**	**YES**

Table S3. Goal 2. Redefining the Role of The WHO Regional Offices.

	Strengthen Regional Competencies	Allocating Direct Financing	Establish National Focal Points	Regional Offices as Support Hubs	Grant Regional Autonomy	Aggregate
Mean	6.37	6.20	6.30	6.40	6.23	6.30

Std Dv	0.96	1.21	1.06	0.67	1.04	0.61
Median	7.00	6.50	7.00	6.50	7.00	6.40
High	7	7	7	7	7	7
Low	3	1	4	5	4	4.8
Top Quartile	7	7	7	7	7	6.8
Bottom Quartile	6	6	6	6	5.5	6
Interquartile Range	1	1	1	1	1.5	0.8
#above or equal to 5	29	29	26	30	27	29
% above or equal to 5	97%	97%	87%	100%	90%	97%
#above or equal to 6	25	25	25	27	23	24
% above or equal to 6	83%	83%	83%	90%	77%	80%
**Strict Consensus Criterion**	**YES**	**YES**	**YES**	**YES**	**YES**	**YES**
**Flexible Consensus Criterion**	**YES**	**YES**	**YES**	**YES**	**YES**	**YES**

Table S4. Goal 3. Securing Sustainable and Equitable Financing.

	Transparent Pandemic Preparedness Fund	Sustainable Financing via Multiple Sources	Priority for Vulnerable Systems	Independent Review Board	Aggregate
Mean	6.00	6.03	6.37	6.37	6.19
Std Dv	1.41	1.22	1.33	1.27	1.24
Median	7.00	6.00	7.00	7.00	6.50
High	7	7	7	7	7
Low	3	3	1	3	3
Top Quartile	7	7	7	7	6.75
Bottom Quartile	6	6	6	6.5	6.13
Interquartile Range	1	1	1	0.5	0.63
#above or equal to 5	25	27	28	27	27
% above or equal to 5	83%	90%	93%	90%	90%
# above or equal to 6	23	24	27	25	24
% above or equal to 6	77%	80%	90%	83%	80%
**Strict Consensus Criterion**	**FALSE**	**YES**	**YES**	**YES**	**YES**
**Flexible Consensus Criterion**	**YES**	**YES**	**YES**	**YES**	**YES**

Table S5. Goal 4. Establishing Equity Monitoring Mechanisms.

	Equity Indicators	Priority to Vulnerable Populations	Public Equity Dashboard	Aggregate
Mean	6.33	6.24	5.93	6.17
Std Dv	1.06	0.99	1.85	0.89
Median	7.00	6.00	7.00	6.33
High	7	7	7	7
Low	3	3	1	3
Top Quartile	7	7	7	6.67
Bottom Quartile	6	6	6	6
Interquartile Range	1	1	1	0.67
#above or equal 5	28	27	25	26
% above or equal to 5	93%	90%	83%	87%
# above or equal to 6	24	25	24	23
% above or equal to 6	80%	83%	80%	77%
**Strict Consensus Criterion**	**YES**	**YES**	**FALSE**	**YES**

Flexible Consensus Criterion	YES	YES	FALSE	YES

Table S6. Goal 5. Adopting Accountability and Monitoring platforms.

	Independent Peer Review	Public Preparedness Score Cards	Real Time Surveillance Dashboard	Mandatory Preparedness Audits	Aggregate
Mean	6.27	5.83	6.43	5.90	6.11
Std Dv	1.17	1.39	0.86	1.30	1.18
Median	7.00	6.00	7.00	6.00	6.50
High	7	7	7	7	7.00
Low	3	2	4	3	3.00
Top Quartile	7	7	7	7	7.00
Bottom Quartile	6	5	6	5	5.50
Interquartile Range	1	2	1	2	1.50
#above or equal to 5	27	26	29	24	26
% above or equal to 5	90%	87%	97%	80%	88%
# above or equal to 6	25	21	25	22	20
% above or equal to 6	83%	70%	83%	73%	78%
Strict Consensus Criterion	YES	FALSE	YES	FALSE	YES
Flexible Consensus Criterion	YES	YES	YES	YES	YES

Table S7. Goal 6. Integrating Health and Non-Health Sectors.

	Partnership Non-Health Sectors	Health in All Policies	Incorporate Climate Health Risk	Aggregate
Mean	6.03	5.87	6.40	6.10
Std Dv	1.04	1.38	1.30	1.24
Median	7.00	6.00	7.00	6.50
High	7	7	7	7
Low	1	1	1	1
Top Quartile	7	7	7	7.0
Bottom Quartile	5	6	6	6
Interquartile Range	2	1	1	1.0
#above or equal to 5	26	26	28	27
% above or equal to 5	87%	87%	93%	90%
# above or equal to 6	21	23	28	23
% above or equal to 6	70%	77%	93%	77%
Strict Consensus Criterion	FALSE	FALSE	YES	YES
Flexible Consensus Criterion	YES	YES	YES	YES

Table S8. Goal 7. Enforcing Legal and Policy Framework (Round 1&2).

	IHR Revision	Binding Equity/Legal Standards	Legal Assistance LMICs/LDCs	Global Treaty Compliance Review	Aggregate
Mean	6.00	6.07	6.33	6.27	6.17
Std Dv	1.41	1.16	1.11	0.80	0.85
Median	7.00	7.00	7.00	6.00	6.50
High	7	7	7	7	7
Low	3	4	4	5	5

Top Quartile	7	7	7	7	7
Bottom Quartile	5.5	5	6	6	5.375
Interquartile Range	1.5	2	1	1	1.625
#above or equal to 5	13	13	13	15	15
% above or equal to 5	87%	87%	87%	100%	100%
# above or equal to 6	11	10	12	12	9
% above or equal to 6	73%	67%	80%	80%	60%
**Strict Consensus Criterion**	**FALSE**	**FALSE**	**YES**	**YES**	**FALSE**
**Flexible Consensus Criterion**	**YES**	**YES**	**YES**	**YES**	**YES**

Table S9. Goal 7. Enforcing Legal and Policy Framework (Round 3).

	**IHR Revision**	**Binding Equity/Legal Standards**	**Legal Assistance LMICs/LDCs**	**Global Treaty Compliance Review**	**Aggregate**
Mean	5.70	5.73	6.37	5.93	6.55
Std Dv	0.51	0.63	0.93	1.01	0.44
Median	6.00	6.00	7.00	6.00	6.50
High	7	7	7	7	7
Low	6	5	4	3	5.75
Top Quartile	7	7	7	7	6.875
Bottom Quartile	5	5	6	6	6.375
Interquartile Range	2	2	1	1	0.5
#above or equal to 5	15	15	15	15	15
% above or equal to 5	100%	100%	100%	100%	100%
# above or equal to 6	15	14	14	13	14
% above or equal to 6	100%	93%	93%	87%	93%
**Strict Consensus Criterion**	**YES**	**YES**	**YES**	**YES**	**YES**
**Flexible Consensus Criterion**	**YES**	**YES**	**YES**	**YES**	**YES**

Table S10. Over-arching Statements.

	**FGGH addresses governance & equity gaps**	**GLOBAL BALANCE**	**LMIC representation**	**cohrent solution**	**Aggregate**
Mean	6.10	6.50	6.50	6.00	6.28
Std Dv	0.99	0.68	0.97	1.05	0.67
Median	6.00	7.00	7.00	6.00	6.50
High	7	7	7	7	7
Low	4	4	3	2	4.25
Top Quartile	7	7	7	7	6.9375
Bottom Quartile	6	6	6	6	6
Interquartile Range	1	1	1	1	0.9375
#above or equal to 5	27	29	28	29	29
% above or equal to 5	90%	97%	93%	97%	97%
# above or equal to 6	23	29	27	23	23
% above or equal to 6	77%	97%	90%	77%	77%
**Strict Consensus Criterion**	**YES**	**YES**	**YES**	**YES**	**YES**
**Flexible Consensus Criterion**	**YES**	**YES**	**YES**	**YES**	**YES**
