# Supplementary material for: Developing and Validating a Global Governance Framework for Health: A Delphi Consensus Study
Source: Int J Environ Res Public Health. 2026 Jan 22;23(1):138. doi: 10.3390/ijerph23010138 (PMC12840802; doi:10.3390/ijerph23010138)
Supplement: Supplementary file 1 [file ijerph-23-00138-s001.zip › File S5 - Table of Expert Consensus Ratings Across Goals.pdf]

Table of Expert Consensus Ratings Across Goals

Goals	Statements	Median	S D	% ≥5	% ≥6	Strict	Flexible
Goal 1: Original Statements (Round 1&2).	Strengthening WHO as the central coordinating body for global pandemic preparedness and response.	7	0.92	83%	93%	YES	YES
	Create a dedicated Global Health Security Coordination Unit to manage future pandemics.	6	1.55	70%	53%	NO	NO
	Establish an UN-WHO Emergency Leadership Authority with a unified command during global health crises.	5	1.59	77%	47%	NO	NO
	Include multilateral institutions and low- and middle-income countries (LMICs) in WHO decision-making processes.	7	0.77	97%	90%	YES	YES
	The Conference of the Parties (COP) should be empowered to review the performance of all stakeholders, including the WHO.	6	0.86	97%	77%	YES	YES
	Aggregate					NO	YES
Goal 1 Modified Statements (Round 3)	Strengthen and clarify WHO's coordinating role in pandemic preparedness by integrating WHE (WHO Health Emergencies Program), SCHEPPR (Independent Oversight and Advisory Committee), and the IOAC (Independent Oversight and Advisory Committee), into a clear structure.	7	0.74	100%	87%	YES	YES
	Develop a dedicated global health security coordination mechanism within WHO by strengthening existing units like WHE (WHO Health Emergencies Program), rather than creating new parallel structures	6	0.8	80%	100%	YES	YES
	Establish a joint UN–WHO Emergency Leadership Authority with a clear coordination mandate and time-bound crisis authority, leveraging existing platforms like the UN Crisis Management Team and WHO Emergency Response Framework.	6	1.03	80%	93%	YES	YES
	Effective pandemic governance should guarantee formal and equitable representation of multilateral institutions (e.g., African Union, ASEAN (Association of Southeast Asian Nations)) and low- and middle-income countries (LMICs) in WHO governance bodies, with meaningful roles in decision-making and agenda-setting.	7	0.59	93%	100%	YES	YES
	The Conference of the Parties (COP) should focus on monitoring state party compliance with the WHO Pandemic Agreement, while the performance review of the World Health Organization (WHO) remains under the authority of the World Health Assembly (WHA), in line with the WHO Constitution.	6	1.26	93%	93%	YES	YES
	Aggregate	6.40	0.58	100%	87%	YES	YES
Goal 2	Strengthen the technical and operational capacities of WHO Regional Offices to ensure they can lead context-specific pandemic responses.	7	0.92	93%	83%	YES	YES

	Allocate direct, flexible financing to WHO Regional Offices to enable rapid and autonomous response during health emergencies.	6.5	1.21	97%	83%	YES	YES
	Establish National Pandemic Preparedness Focal Points in every country to provide surge capacity technical assistance, and policy guidance to countries.	7	1.06	87%	83%	YES	YES
	Position Regional Offices as operational support hubs that provide surge capacity, technical assistance, and policy guidance to countries.	6.5	0.67	100%	90%	YES	YES
	Provide WHO Regional Offices with greater administrative and operational autonomy while maintaining alignment with global governance standards.	7	1.04	90%	77%	YES	YES
	Aggregate	6.4	0.61	97%	80%	YES	YES
Goal 3	A well-governed, transparent Global Pandemic Preparedness Fund, established as a permanent mechanism rather than a crisis-driven response, is important for ensuring sustainable and equitable financing for health security at all times.	7	1.41	83%	77%	NO	NO
	The Global Pandemic Preparedness Fund should ensure sustainable, equitable financing, supported by member states, the private sector, and levies on high-risk industries.	6	1.22	90%	80%	NO	NO
	Allocation criteria should explicitly prioritize countries with fragile health systems and regions most vulnerable to future pandemics.	7	1.33	93%	90%	YES	YES
	The fund should be overseen by an independent review board with transparent reporting and representation from LMICs.	7	1.27	90%	83%	NO	NO
	Aggregate	6.5	1.24	90%	80%	YES	YES
Goal 4	Equity considerations must guide emergency response efforts, with priority given to vulnerable populations and under-resourced health systems.	7	1.06	93%	80%	YES	YES
	Developing a Pandemic Preparedness Equity Index can support transparent and data-informed resource allocation	6	0.99	90%	83%	YES	YES
	Equity outcomes should be regularly tracked and published through a global, publicly accessible dashboard to ensure accountability.	7	1.85	83%	80%	NO	NO
	Aggregate	6.33	0.89	87%	77%	YES	YES
Goal 5	Regular, independent peer reviews of national and regional pandemic preparedness capacities are important for transparency and continuous improvement.	7	1.17	90%	83%	YES	YES
	Publishing standardized preparedness scorecards will support comparative evaluation and public accountability.	6	1.39	87%	70%	NO	YES
	A real-time Global Disease Surveillance Dashboard, coordinated by WHO, will strengthen early warning systems and response readiness.	7	0.86	97%	83%	YES	YES

	Countries are mandated to publish preparedness audits and response assessments as part of treaty compliance and international transparency obligations.	6	1.3	80%	73%	NO	YES
	Aggregate	6.5	1.18	88%	78%	YES	YES
Goal 6	Formal partnerships between WHO and non-health sectors (e.g., FAO, UNEP, WTO) are important to addressing upstream pandemic drivers.	7	1.04	87%	70%	NO	YES
	Institutionalizing a "Health in All Policies" (HiAP) approach will ensure health considerations are embedded across sectors such as trade, education, housing, and environment.	6	1.38	87%	77%	NO	YES
	Pandemic preparedness must incorporate climate-related health risks, to address emerging cross-sectoral health threats.	7	1.3	93%	93%	YES	YES
	Aggregate	6.5	1.24	90%	77%	YES	YES
Goal 7 Original Statements (Round 1&2).	The International Health Regulations (IHR) must be revised to impose binding, enforceable obligations for pandemic preparedness and response.	6	1.51	83%	63%	NO	NO
	The effectiveness of the WHO Pandemic Agreement (2025) depends on the adoption of binding provisions and equity-driven legal standards that apply uniformly across high, middle, and low-income countries.	6	1.2	80%	63%	NO	YES
	WHO and relevant international bodies should provide technical and legal assistance to countries, particularly LMICs and LDCs, to support the drafting, revision, and enforcement of national legislation that aligns with treaty obligations.	7	0.93	93%	83%	YES	YES
	A global treaty compliance review mechanism should be created to conduct regular legal audits of national pandemic laws and policies, benchmarked against the IHR and the Pandemic Agreement.	7	1.01	90%	77%	YES	YES
	Aggregate	6	0.84	90%	60%	NO	YES
Goal 7 Modified Statements (Round 3)	The 2024 amendments to the International Health Regulations (IHR) should be the primary legal framework for pandemic preparedness, with a focus on supporting national compliance through technical and financial assistance.	7	0.51	100%	100%	YES	YES
	The WHO Pandemic Agreement should embed binding obligations based on the principle of Common but Differentiated Responsibilities (Common but Differentiated Responsibilities (CBDR) is a principle recognizing that while all states share responsibility for global challenges, their obligations should reflect differences in national capacity (e.g., financial, technical, and institutional resources) and historical contribution to the problem (e.g., actions or inactions that have exacerbated global risks).) (CBDR), ensuring equitable commitments tailored to national capacity.	7	0.63	100%	93%	YES	YES

	The WHO, in coordination with regional institutions (e.g., African Union, ASEAN), should provide sustained technical and legal support to low- and middle-income countries and least developed countries to align national legislation with treaty obligations.	7	0.63	100%	93%	YES	YES
	A cooperative and transparent treaty compliance review mechanism should be established, building on existing tools such as JEE (Joint External Evaluation reporting), SPAR (State Party Self-Assessment Annual Reporting), and regional peer-review platforms to promote mutual accountability.	7	0.74	100%	87%	YES	YES
	Aggregate	6.50	0.40	100%	93%	YES	YES
Goal 8	The proposed Framework for Global Governance for Health (FGGH) addresses the institutional, equity, and coordination gaps exposed during the COVID-19 response and those partially unresolved by the 2025 WHO Pandemic Agreement.	6	0.99	90%	77%	YES	YES
	Balancing global coordination with respect for national sovereignty is a core challenge in implementing the FGGH.	7	0.68	97%	97%	YES	YES
	Ensuring robust and equitable representation of LMICs in decision-making is important to building legitimacy and accountability.	7	0.97	97%	77%	YES	YES
	The seven goals of the FGGH offer a coherent, actionable roadmap to strengthen global pandemic preparedness.	6	1.05	97%	77%	YES	YES
	Aggregate	6.5	0.67	97%	77%	YES	YES
